# *Brca1 *breast tumors contain distinct CD44^+^/CD24^- ^and CD133^+ ^cells with cancer stem cell characteristics

**DOI:** 10.1186/bcr1855

**Published:** 2008-02-01

**Authors:** Mollie H Wright, Anna Maria Calcagno, Crystal D Salcido, Marisa D Carlson, Suresh V Ambudkar, Lyuba Varticovski

**Affiliations:** 1 Laboratory of Human Carcinogenesis, Center for Cancer Research, National Cancer Institute, 9000 Rockville Pike, Bethesda, Maryland 20892, USA; 2 Laboratory of Cell Biology, Center for Cancer Research, National Cancer Institute, 9000 Rockville Pike, Bethesda, Maryland 20892, USA

## Abstract

**Introduction:**

Whether cancer stem cells occur in *BRCA1*-associated breast cancer and contribute to therapeutic response is not known.

**Methods:**

We generated and characterized 16 cell lines from five distinct *Brca1deficient *mouse mammary tumors with respect to their cancer stem cell characteristics.

**Results:**

All cell lines derived from one tumor included increased numbers of CD44^+^/CD24^- ^cells, which were previously identified as human breast cancer stem cells. All cell lines derived from another mammary tumor exhibited low levels of CD44^+^/CD24^- ^cells, but they harbored 2% to 5.9% CD133^+ ^cells, which were previously associated with cancer stem cells in other human and murine tumors. When plated in the absence of attachment without presorting, only those cell lines that were enriched in either stem cell marker formed spheroids, which were further enriched in cells expressing the respective cancer stem cell marker. In contrast, cells sorted for CD44^+^/CD24^- ^or CD133^+ ^markers lost their stem cell phenotype when cultured in monolayers. As few as 50 to 100 CD44^+^/CD24^- ^or CD133^+ ^sorted cells rapidly formed tumors in nonobese diabetic/severe combined immunodeficient mice, whereas 50-fold to 100-fold higher numbers of parental or stem cell depleted cells were required to form few, slow-growing tumors. Expression of stem cell associated genes, including *Oct4*, *Notch1*, *Aldh1*, *Fgfr1*, and *Sox1*, was increased in CD44^+^/CD24^- ^and CD133^+ ^cells. In addition, cells sorted for cancer stem cell markers and spheroid-forming cells were significantly more resistant to DNA-damaging drugs than were parental or stem cell depleted populations, and they were sensitized to the drugs by the heat shock protein-90 inhibitor 17-DMAG (17-dimethylaminoethylamino-17-demethoxygeldanamycin hydrochloride).

**Conclusion:**

*Brca1*-deficient mouse mammary tumors harbor heterogeneous cancer stem cell populations, and CD44^+^/CD24^- ^cells represent a population that correlates with human breast cancer stem cells.

## Introduction

*BRCA1 *was the first identified breast cancer susceptibility gene and was localized to 17q21 by positional cloning more than 15 years ago [1]. *BRCA1 *is mutated in about 2.5% to 5% of all breast cancers, in 45% of inherited breast cancer families, and in up to 80% of breast/ovarian cancer families. *BRCA1 *mutation is associated with a high incidence of bilateral disease, and confers an 82% risk for developing breast cancer and an 54% risk for developing ovarian cancer by age 80 years [2]. Somatic mutations of *BRCA1 *have been reported in up to 10% of cases of sporadic ovarian cancer, but they are extremely rare in sporadic breast cancer [3-5]. However, reduced BRCA1 protein expression is detected in high-grade sporadic breast and ovarian tumors, suggesting that epigenetic downregulation of *BRCA1 *contributes to their aggressive clinical course [6-8]. The existence of cancer stem cells associated with *BRCA1 *mutations or downregulation has not been reported.

In spite of early detection and aggressive surgical and chemotherapeutic approaches, no significant 5-year survival benefits have been achieved in patients harboring *BRCA1 *mutations [6]. During the past several years, cancer stem cells have been subjected to increasing scrutiny as a potential cause of relapse and drug resistance [9]. Several groups [10,11] identified a small subpopulation of highly tumorigenic cells from human breast tumors bearing the CD44^+^CD24^-/low ^lineage phenotype, which have drug-resistant phenotype and the capacity to form tumors after transplantation in nonobese diabetic/severe combined immunodeficient mice. Subsequent enrichment in Sca-1 positive cancer stem cells was shown for mouse mammary tumor models, such as mouse mammary tumor virus (MMTV)-Her2/neu and MMTV-Wnt1 [12], and Thy1/CD24 expression further defined cancer stem cells in the Wnt1 model [13]. No studies have yet been conducted to characterize *Brca1*-deficient cancer stem cells.

Multiple mouse models with targeted deletion of *Brca1 *in the mammary gland generate tumors with low penetrance [14]. Increased incidence of these tumors is observed in mice harboring two *Brca1*^Δ*exon*11 ^genes in a *p53*^+/- ^background, with uniform deletion of p53 in these tumors. Lymphomas were also reported in this model [15]. However, the *Brca1 *deficient mouse mammary tumors have variable penetrance and latency, which makes it nearly impossible to use these models to standardize therapies and to study the stem cell population. To overcome these difficulties, we developed and characterized 16 cell lines from five independent *Brca1*^Δexon11^/*p53*^+/- ^tumors. We examined these cell lines for specific cell populations using multiple known stem cell markers. Cell populations expressing putative stem cell markers were more resistant to chemotherapeutic agents than were parental cells, and had other characteristics of cancer stem cells, including reconstitution of tumors by as few as 50 to 100 cells.

## Materials and methods

### Generation of cell lines from *Brca1 *mouse mammary tumors

*Brca1 *tumor cell suspensions were prepared as described by Varticovski and coworkers [16] from *Brca1*^Δ11^*p53*^+/- ^mammary tumors. Briefly, mice were euthanized with CO_2_, and tumors were collected aseptically and mechanically dissociated. Cells were passaged through a 40 μm mesh screen, and were further dissociated by serial passage through a syringe with 18 to 25 gauge needles. Cells were plated at low density for selection of individual clones. Cells were grown at 37°C in 5% carbon dioxide in RPMI 1640 media supplemented with penicillin/streptomycin, glutamine, and fetal bovine serum starting with 2% and progressively increasing to 10%. More than 40 clones were isolated using cloning cylinders and a total of 16 cell lines were developed from five independent primary tumors. Each cell line was passaged weekly and maintained in culture for up to 50 passages.

### Characterization of unsorted cells that survive in long-term cultures in the absence of attachment

The ability of cells to grow in the absence of attachment was tested by plating cells on low-binding plates (Fisher Scientific, Hampton, NH, USA). Under these conditions, some cell lines showed surviving floating colonies that formed compact spheroids after 3 to 4 weeks of culture. Spheroid formation frequency for each cell line was tested by plating cells in limiting dilution from 500 to 1 cell/well on 96-well low-binding plates in sextuplicate. The number of colonies was scored weekly, and the spheroids were dispersed into single cells and upstaged to six-well plates after 3 weeks. To obtain single cell suspension, spheroids were collected and washed in phosphate-buffered saline (PBS). Single cell suspension was obtained by incubation in collagenase/dispase and DNAse (Roche, Switzerland), filtering through a 40 μm filter to remove remaining aggregates (Fisher Scientific), and plated directly onto 96-well tissue culture plates for drug testing or passaged onto a new low-binding plate for expansion.

### Analysis of cell surface markers

Cell lines grown as monolayer were trypsinized, and cells grown as spheroids were dispersed to obtained single cell populations, as above. Cells were washed in PBS with 1% bovine serum albumin and stained with PE anti-mouse CD24 (BD Pharmingen, San Jose, CA, USA), or APC anti-mouse CD24 (Biolegend, San Diego, CA, USA), FITC anti-mouse CD44 (Southern Biotech, Birmingham, AL, USA), or PE anti-mouse prominin-I CD133 (eBioscience, San Diego, CA, USA). Rat IgG (CHEMICON, Billerica, MA, USA) was used as the isotype control, in accordance with the manufacturer's instructions. Cells were analyzed by flow cytometry using LSR II (BD Biosciences, San Jose, CA, USA). Data were collected using FACSDiVa software (BD Biosciences) from no fewer than 30,000 cells.

### Cytotoxicity assay and calculation of combination index

Doxorubicin was obtained from Sigma (St. Louis, MO, USA) and dissolved in PBS as 10 mmol/l stock. Aliquots were frozen and diluted in media immediately before use. Cisplatin and etoposide were obtained from Sigma-Aldrich (St. Louis, MO, USA). 17-DMAG (17-dimethylaminoethylamino-17-demethoxygeldanamycin hydrochloride) was obtained from Invivogen (San Diego, CA, USA). These drugs were dissolved in dimethyl sulfoxide, stored as 10 mmol/l stock aliquots, and diluted in media immediately before use.

Cells were seeded in six replicates in 96-well plates at 10,000 cells per well. Serial dilutions of doxorubicin, cisplatin, etoposide, and 17-DMAG in medium were added to the cells on the following day or as indicated. Dose-response curves to single drugs at 24 hours, 48 hours, and 72 hours after exposure were generated to determine the range of concentrations to be used in combination. For drug interaction studies, drugs were added sequentially (with the 17-DMAG introduced with a 24-hour delay) or simultaneously in a final volume of 100 μl. Cytotoxicity was measured using the Cell Titer 96 Aqueous One Solution Cell Proliferation Assay (Promega, Madison, WI, USA), which is a colorimetric method for determining the number of viable cells based on bioreduction of the tetrazolium compound MTS (3-[4,5-dimethylthiazol-2-yl]-5-[3-carboxymethoxyphenyl]-2-[4-sulfophenyl]-2*H*-tetrazolium, inner salt) by metabolically active cells. After exposure to a single drug or after a total of 48 hours in sequential addition experiments, 20 μl MTS reagent was added to each well and the plates were incubated in a humidified 37°C incubator with 5% carbon dixoide for 1 to 4 hours. Absorbance at 490 nm was recorded using a 96-well plate reader. Data were averaged and normalized against the average survivals of untreated samples and analyzed using CalcuSyn (Biosoft, Ferguson, MO, USA), software based on the multiple drug-effect equation of Chou and Talalay [17].

### Tumor growth *in vivo*

All studies were conducted in an AAALAC (Association for Assessment and Accreditation of Laboratory Animal Care International) accredited facility, in compliance with the US Public Health Service guidelines for the care and use of animals in research. Parental cells and cells sorted for indicated cell surface markers were resuspended in 100 μl RPMI media and injected into the inguinal mouse fat pad (pad #4) of 6- to 8-week-old female nonobese diabetic/severe combined immunodeficient mice. The growth of tumors was monitored using caliper measurements to determine tumor mass. Weights (milligrams) were calculated from measurements (millimeters) of two perpendicular dimensions (length and width) using the formula for a prolate ellipsoid and assuming a specific gravity of 1.0 mg/mm^3 ^[18].

### RNA extraction and analysis of stem cell-associated genes

Total RNA was isolated using TRIzol reagent (Invitrogen, Carlsbad, CA, USA), in accordance with the manufacturer's instructions, followed by DNAse treatment and RNA clean-up (RNeasy Mini Kit; Qiagen, Valencia, CA, USA). RNA concentration and integrity was determined using the RNA 6000 Nano LabChip Kit (Qiagen) on Agilent 2100 Bioanalyzer.

The mRNA levels of 84 genes associated with stem cell biology were examined using human Stem Cell RT^2 ^profiler arrays (SuperArray Bioscience, Frederick, MD, USA), in accordance with the manufacturer's instructions. RNA (250 to 500 ng) was reverse transcribed using the First Strand Synthesis Kit (Qiagen), and cDNA was subjected to real-time PCR using SYBR green/ROX Master Mix (Qiagen) and PCR cycles were performed on a 7500 Real Time PCR System (Applied Biosystems, Foster City, CA, USA). A dissociation curve was run as a quality control using default melting curve settings. Values obtained for the threshold cycle for each gene were normalized using the average of housekeeping genes amplified on the same array.

### Real-time quantitative RT-PCR

Real-time quantitative RT-PCR was used to measure mRNA expression levels of selected mouse ATP-binding cassette (ABC) transporters using LightCycler RNA Master SYBR Green Kit and the LightCycler 480 instrument (Roche Biochemicals, Indianapolis, IN, USA). Specific PCR primer sequences for genes are listed in Additional file 1. All RT-PCR reactions were performed on 200 to 400 ng total RNA with 250 nmol/l specific primers. Negative controls consisting of no-template (water) reaction mixtures were run with all reactions. Melting curves were determined for each primer set following all RT-PCR runs using the LightCycler 480 software. Crossing points for each transcript were determined using the second derivative maximum analysis with the arithmetic baseline adjustment. Crossing point values for each transporter were normalized to the respective crossing point values for reference gene *Pmca4 *(plasma membrane calcium ATPase 4) [19]. Data are presented as fold change in gene expression using the ΔΔCt method.

### Immunostaining

Cells growing as spheroids were expanded in low-binding six-well plates, collected, allowed to attach over 2 hours to a multiwell chamber coated with D-poly L-lysine, fixed in 4% paraformaldehyde, and permeabilized with 0.3% Triton X-100 in PBS (15 minutes) and washed in PBS. Cells were blocked in donkey serum for 1 hour, and incubated with anti-Numb, anti-Oct4, anti-Nestin, or anti-CD133 primary antibodies overnight (Abcam, Cambridge, MA, USA). Secondary antibodies conjugated to Alexa Fluor 488 and Alexa Fluor 568 (Molecular Probes, Eugene, OR, USA) were added for 1 hour, washed in PBS, and mounted under a coverslip using Vectashield Mounting Medium (Vector Laboratories, Burlingame, CA, USA). Immunofluorescence was visualized in an Olympus 1X51 fluorescence microscope (Olympus America Inc., Center Valley, PA, USA.

## Results

### Characterization of cell lines from *Brca1 *mouse mammary tumors

Sixteen cell lines were generated from five independent original *Brca1*^Δexon11^/*p53*^+/- ^mouse mammary tumors [15,16]. These cells had similar doubling time of approximately 18 to 20 hours and similar morphology with epithelioid appearance, although A1-derived cell lines had some rounded cells (Additional file 2). As previously described for *Brca1 *tumors in this model [15], all original tumors and cell lines expressed mutant *Brca1*^Δexon11 ^and lacked *p53*, as determined by PCR (data not shown). Several cell lines representing each one of the five original tumors were selected at random for additional studies.

**Figure 1:**
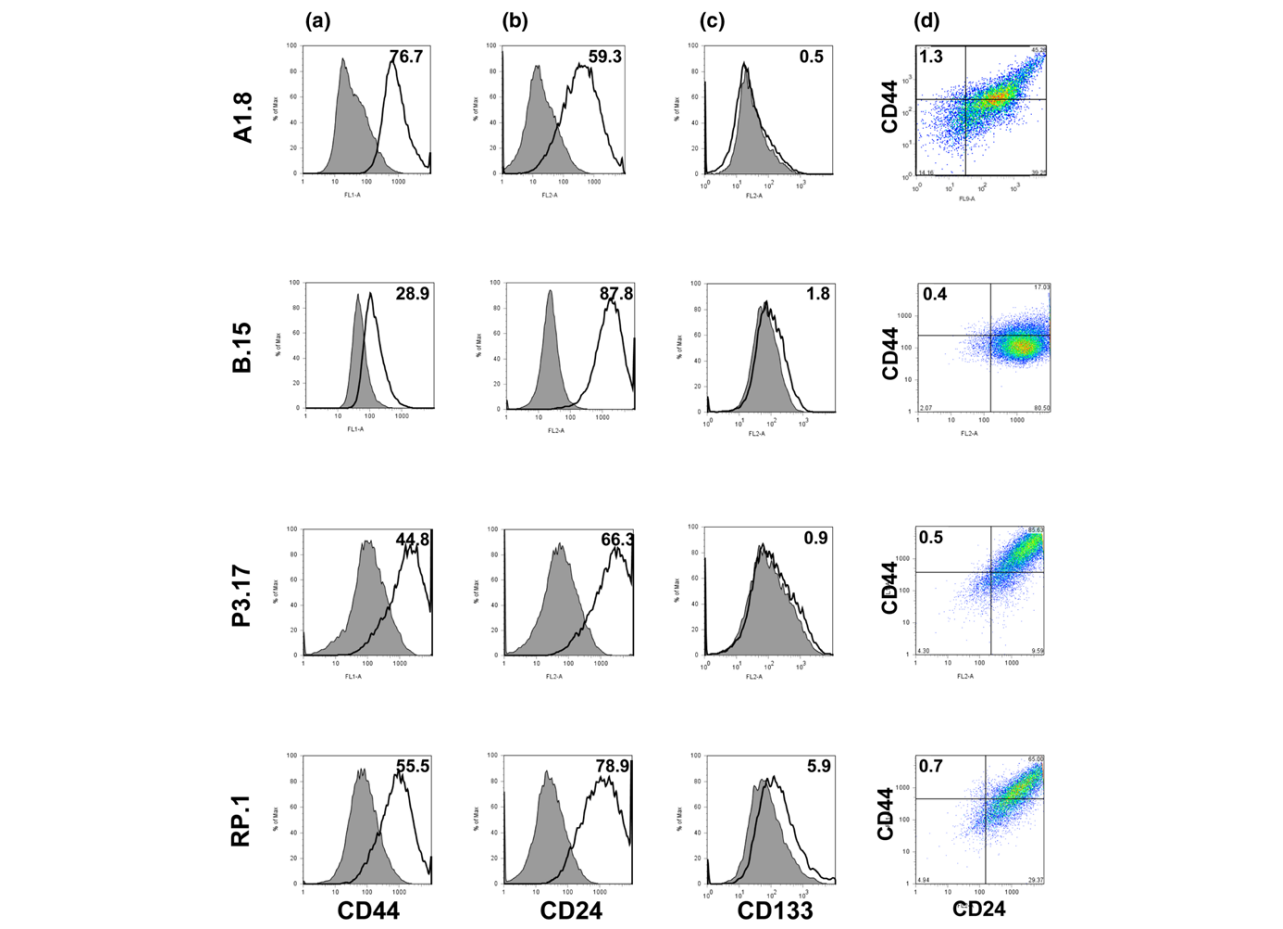
Expression of putative stem cell markers from cells derived from five individual *Brca1 *tumors. **(a) **Cell surface expression of CD44 is relatively uniform across all cell lines. The open histograms, outlined by a black line, represent positive staining for CD44, and gray filled histograms show negative controls stained with matched isotype antibody. **(b) **Expression of CD24 is variable, with highest level in B.15 cells. **(c) **There was substantial expression of CD133^+ ^cells, accounting for 5.9% of the total population, among RP.1 cells. **(d) **Co-expression of CD44 and CD24 markers. The quadrants are gated to separate double positive, single positive, and double negative populations. The percentage of cells with CD44^+^/CD24^- ^markers is indicated at the upper left of each panel. The highest fraction is in cells derived from A1 tumor, as represented by A1.8 cells. One of more than three independent experiments is shown here.

To determine whether *Brca1 *cell lines contain a distinct population of cancer stem cells, we examined expression of cell surface markers previously assigned to human breast cancer stem cells, namely CD44 and CD24. All *Brca1 *clones expressed CD44 to varying degrees, with the B.15 cell line expressing the lowest percentage of positive cells (Figure 1a). Expression of CD24 was also similar among the cell lines, but higher in B.15 cells (Figure 1b). All cell lines derived from A1 tumor (A1.1, A1.8, and A1.10) exhibited a higher (1.32% to 5%) fraction of CD44^+^/CD24^- ^cells, which correlate with the phenotype of human breast cancer stem cells (Figure 1d).

We also tested whether *Brca1 *cells express CD133, a marker not previously described in association with breast cancer, but shown to mark cancer stem cells in other tumors. Only RP tumor-derived cell lines had a significant CD133^+ ^population, which varied between 2% and 5.9%, as shown for RP.1 cell line (Figure 1c). All other cell lines had low CD133^+ ^population. There was no overlap between CD133^+ ^and CD44^+^/CD24^- ^populations, as determined by analysis of cells after triple staining (Additional file 3 [panels A and B]). Thus, individual *Brca1*-deficient mouse mammary tumors gave rise to cell lines with distinct populations of cells expressing stem cell markers. The studies described below address differences and similarities between these putative cancer stem cell populations, as defined by these two types of cell surface markers.

### Cells that express stem cell markers survive and proliferate as spheroids in long-term cultures in the absence of attachment

**Figure 2:**
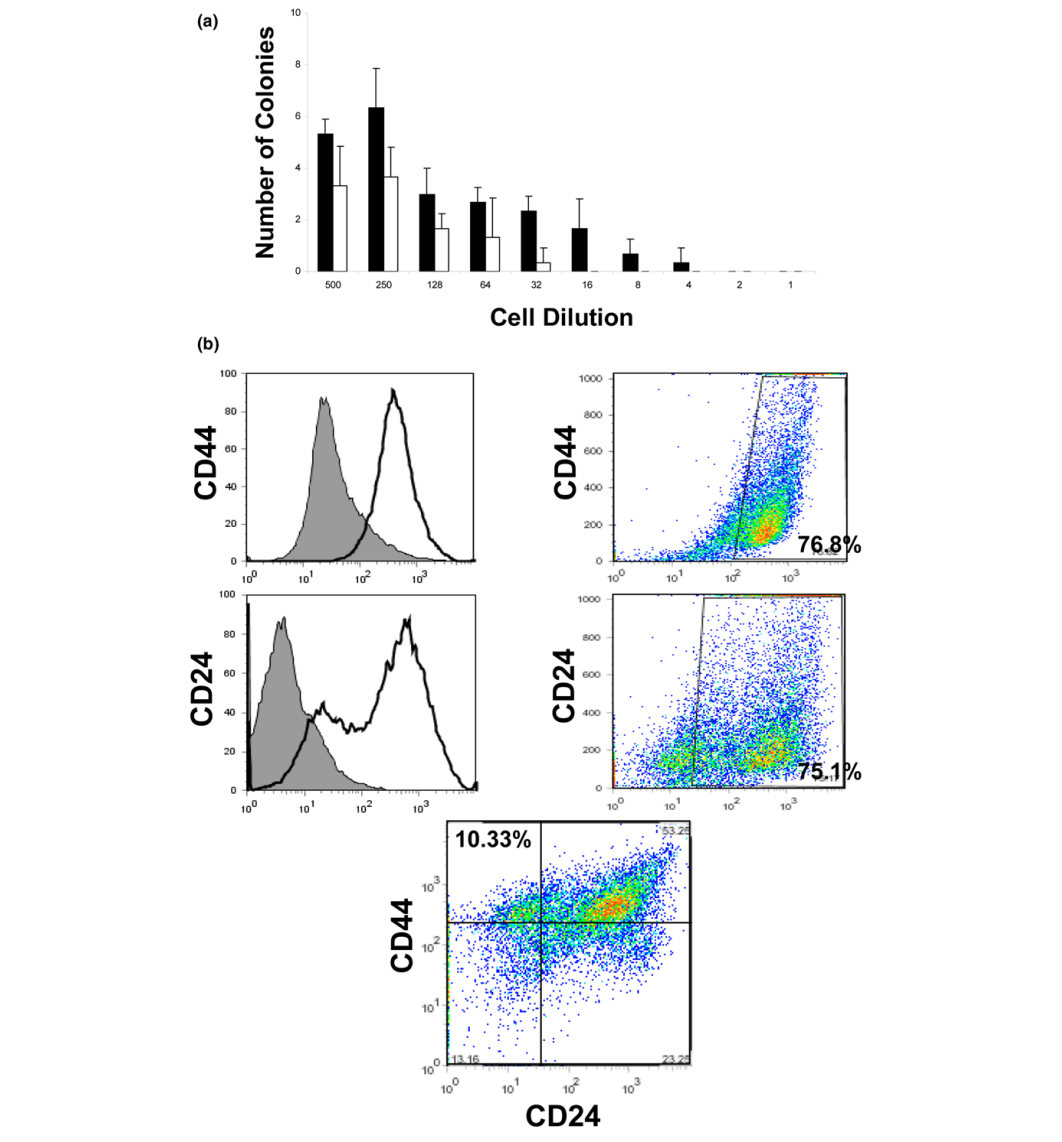
Cells that express stem cell markers survive and form spheroid structures in the absence of attachment. **(a) **Frequency of spheroid formation is increased in *Brca1 *A1.8 cells sorted for SC^+ ^(CD44^+^/CD24^-^) as compared with SC^- ^(CD24^+^/CD44^-^) populations. The results shown are from long-term spheroid assay performed by limiting dilution. The black bars represent numbers of spheroids formed by CD44^+^/CD24^- ^sorted cells/well and the white bars represent numbers from CD24^+^/CD44^- ^cells ± standard deviation from six replicate wells at the end of 2 weeks in culture. The numbers on the ordinate show the number of cells plated/well. One of three independent experiments is shown. **(b) **Unsorted *Brca1 *A1.8 cells plated in the absence of attachment form spheroids, which are enriched in cells expressing stem cell markers. A1.8 were plated as single cells in low binding plates for 2 to 3 weeks, and the resulting spheroids were expanded for 4 subsequent passages, dispersed into single cell population, and stained with appropriate antibodies (as described in Materials and methods). The CD44 and CD24 markers are shown as an open histogram on the left, and filled histograms show negative controls stained with matched isotype antibody. The single stain analysis is on the right panel; the double staining is shown on the bottom. Results are presented as percentage of CD44^+^/CD24^- ^cells from the total population. Note the appearance of a dual population that contains CD24^-/*Low *^cells not evident in parental cells run side-by-side with the spheroid-derived cells and illustrated for the parental A1.8 cell line in Figure 1. One representative experiment from more than three is shown here.

A characteristic of cancer stem cells is their ability to grow in three-dimensional structures in semisolid support or in liquid culture in the absence of attachment, termed spheroids or mammospheres. To determine the capacity of *Brca1 *cells to form spheroids in the absence of attachment, we sorted A.8 cells for CD44^+^/CD24^- ^cells (SC^+^) and CD44^-^/CD24^+ ^(SC^-^) populations. Cells were plated by limiting dilution from 500 to 1 cell/well in six replicate wells in low-binding 96-well plates. Over 2 to 3 weeks, some cells and cell aggregates were formed but degenerated, but many survived and formed tight actively growing spheroids. The SC^+ ^population exhibited a significantly higher number of proliferating viable spheroids, as compared with the SC^- ^population, and generated spheroids even when plated as a low as four to six cells/well (Figure 2a). To determine whether these spheroids can be expanded *in vitro*, the spheroids were dissociated into single cell suspensions and passaged multiple times in long-term sphere-forming assay. These cells repeatedly formed spheroids for up to eight subsequent passages when plated in the absence of attachment. Thus, the A1.8 cell line contains a population of cells that survives in the absence of attachment and forms spheroids that can be expanded *in vitro*.

### Spheroids formed from unsorted cells are enriched in cells which express cancer stem cell markers

To determine the expression of putative cancer stem cell markers in the spheroids, we plated A1.8 and RP.1 cell lines without prior sorting as single cells/well in low-binding 96-well plates. The frequency of spheroid formation was 2% to 4%, as expected from the fraction of cells expressing respective cancer stem cell markers in these cell lines. The resulting spheroids were dissociated into single cells and expanded in 96-well and then in six-well low-binding plates. Analysis of cells derived from spheroids after multiple passages showed spontaneous enrichment in cells expressing putative cancer stem cell markers. In addition, a distinct subpopulation of cells was evident, namely a CD24^-/low ^population (Figure 2b), which was not previously observed in any of the parental cell lines (Figure 1b and data not shown). More than 10% of cells derived from expanded A1.8 spheroids acquired a CD44^+^/CD24^- ^phenotype (Figure 2b, lower panel) and more than 30% were CD44^+^/CD24^-/low^. Similar observations were made for RP.1 cells, in which spheroids had 27.7% CD133^+ ^cells after multiple passages (Additional file 4).

To determine whether any of the other *Brca1 *cell lines can grow in the absence of attachment, we plated each of the 16 cell lines in long-term spheroid-forming assay in limiting dilution, and as single cells/well. Cells were monitored for growth and the number of spheroids was scored after 2 to 3 weeks in culture. Cell morphology varied greatly, as shown for A1.1, A1.8, B.15, P3.17, P2.1, and RP.1 cells after 2 weeks in culture (Additional file 5). Cell lines derived from B, P2, and P3 tumors had small aggregates and floating nonviable cells. In contrast, all cell lines from A1 and RP.1 tumors had viable spheroids in 96-well cultures, and proliferated vigorously as spheroids after subsequent multiple passages *in vitro*. Coincidently, these cell lines also exhibited a higher fraction of cells in CD44^+^/CD24^- ^or CD133^+ ^populations, respectively. Because other cell lines failed to grow as spheroids, we did not attempt to characterize the minute fraction of cells that express CD44^+^/CD24^- ^markers in those cell lines.

### Cells sorted for putative stem cell markers repopulate the parental cell fractions after few passages in monolayer culture

We examined whether cells sorted for positive or negative expression, or whether the putative stem cell makers maintain their phenotypes when grown as monolayers in conventional tissue culture dishes. A1.8 cells were sorted as 100% CD44^+^/CD24^- ^(SC^+^) or CD44^-^/CD24^+ ^(SC^-^) populations and plated as monolayer. Only 2.7% of SC^+ ^cells retained CD44^+^/CD24^- ^phenotype after three passages, thus reconstituting the 1.32% to 5% fraction found in parental cells (Additional file 6 [panel A] and Figure 1). Furthermore, cells sorted from the opposite quadrant for CD44^-^/CD24^+ ^phenotype continued to be depleted in CD44^+^/CD24^- ^markers (0.05%). Thus, expansion of CD44^+^/CD24^- ^cells in monolayer leads to reconstitution of both CD44^+^/CD24^- ^and CD44^-^/CD24^+ ^cell fractions found in parental cells, whereas cells depleted of CD44^+^/CD24^- ^cells were unable to repopulate the CD44^+^/CD24^- ^fraction.

Similarly, RP.1 cells sorted as 100% CD133^+ ^cells exhibited decreased expression after two passages in monolayer (Additional file 6 [panel B]), whereas cells depleted of the CD133^+ ^population (sorted as 100% CD133^-^) were unable to reconstitute the parental population and had a low fraction (0.6%) of CD133^+ ^cells. These data reveal that CD44^+^/CD24^- ^and CD133^+ ^cells have similar capacity for self-renewal and repopulate cell fractions found in the respective parental cells.

### *Brca1 *cells have variable sensitivity to DNA damaging agents

To establish the sensitivity of *Brca1 *cell lines to different drugs frequently used to treat breast cancer patients, cell lines were treated with different classes of DNA-damaging agents represented by doxorubicin, cisplatin, and etoposide. In addition, we used a novel orally available molecular targeted agent, namely 17-DMAG, which is a naturally occurring ansamycin antibiotics derived from geldanamycin (GA, NSC 122750). 17-DMAG was tested because it affects multiple targets and interferes with both cell survival and DNA repair pathways [20-24]. All cells were relatively similar in their response to cisplatin, with a 50% inhibitory concentration (IC_50_) of 3 to 8 μmol/l, and were variably sensitive to other agents (Additional file 7 and data not shown).

### Cells expressing stem cell markers and cells derived from spheroids are highly resistant to chemotherapeutic agents

**Figure 3:**
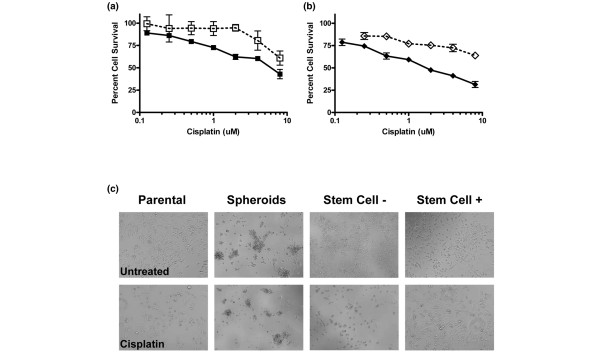
*Brca1 *cells expressing stem cell markers and cells growing as spheroids are highly resistant to cisplatin. A1.8 parental cells, cells from dispersed spheroids, and cells sorted for Stem Cell + (CD44^+^/CD24^-^) and Stem Cell – (CD44^-^/CD24^+^) markers were treated simultaneously with increasing concentrations of cisplatin for 48 hours. **(a) **Parental A1.8 cells (solid symbols) compared with spheroid-derived cells (open symbols). **(b) **A1.8 cells sorted as Stem Cell + (open symbols) and Stem Cell – (solid symbols) populations. **(c) **Morphologic appearance of control untreated cells and cells exposed to 8 μmol/l cisplatin, as indicated on each panel. Note the formation of aggregates from spheroid-derived dispersed cells at the end of 72 hours in culture. One of three independent experiments is shown here.

We compared sensitivity to drugs of parental A1.8 cells, cells dissociated from spheroids, and cells sorted for CD44^+^/CD24^- ^(SC^+^) or CD44^-^/CD24^+ ^(SC^-^) populations plated immediately after sorting. Cells were treated side-by-side on the following day with increasing concentrations of cisplatin (Figure 3). Cells derived from spheroids were significantly more resistant to cisplatin than parental cells, with IC_50_s of 16.675 μmol/l and 4.274 μmol/l, respectively (Figure 3a). In addition, the SC^- ^(CD44^-^/CD24^+^) population was significantly more sensitive than was the parental or SC^+ ^(CD44^+^/CD24^-^) populations, with an IC_50 _of 1.384 μmol/l. Furthermore, CD44^+^/CD24^- ^(SC^+^) cells had an estimated IC_50 _of 45.846 μmol/l (Figure 3b), as calculated using CalcuSyn Software. Because of gradual loss of stem cell markers in cells growing as a monolayer, these data may further underestimate chemoresistance of the cancer stem cells. The morphology of each cell fraction after 48 hours of exposure to 8 μmol/l cisplatin is shown in Figure 3c. Although the parental and SC^- ^population exhibited significant signs of toxicity, SC^+ ^cells have few signs of toxicity and cells dispersed from spheroids showed small aggregates, indicating a delay in cell growth, but remarkably there were no significant signs of toxicity. Similar results were obtained with parental RP.1 cells, spheroids, and cells sorted for CD133^+ ^and CD133^- ^populations (data not shown).

### Cells sorted for cancer stem cell makers lose their drug resistance when grown in monolayers

To determine whether cells sorted for putative cancer stem cell markers lose the drug resistance after growing in monolayers, sorted A1.8 CD44^+^/CD24^- ^(SC^+^), CD44^-^/CD24^+ ^(SC^-^), and RP.1 CD133^+ ^and CD133^- ^cells were passaged four times as monolayer. In addition to losing enrichment in stem cell markers, as described above, these cells lost differences in sensitivity to cisplatin as compared with parental cells (data not shown).

### Expression of multidrug resistance genes in *Brca1 *stem cells

To determine whether drug resistance of cells expressing stem cell surface markers is due to expression of multidrug resistance genes, we compared expression of several selected ABC transporters in five cell lines representative from each primary tumor and compared their expression with normal mammary gland from C57/Bl6 mice. Data were normalized to expression of *Pmca4*, a housekeeping gene [19]. Using a panel of ABC transporters most likely to be responsible for drug resistance to the chemotherapeutic agents tested, we included *Abcb1a*, *Abcb1b*, *Abcc1*, and *Abcg2*. Expression of *Abcg2 *was higher in all cell lines except for P3.17. The highest level, with a 25-fold increase, was in B.15 cells, as compared with expression in normal mammary gland (C57/Bl6; Additional file 8 [panel A]). Thus, *Abcg2 *expression did not correlate with the presence of stem cell population or with the ability of cells to survive in the absence of attachment, because B15 cells were more sensitive to all drugs, lacked enrichment in stem cell markers, and did not form spheroids.

Remarkably, higher *Abcb1b *(*Pgp*, *Mdr1*) expression, but not that of the other ABC transporters, was detected in cell lines enriched in cancer stem cell markers, namely A1.8 and RP.1 (Additional file 8 [panel B] and data not shown).

We also compared expression of ABC transporters in parental cell lines, spheroids, and cells sorted for positive or negative stem cell markers, versus RNA levels in the respective original A1 and RP *Brca1 *tumors (Additional file 8 [panels C and D]). For *Abcb1b*, parental and sorted populations of A1.8 cells had a substantial fivefold to ninefold increase in *Abcb1 *expression, with the greatest increase in A1.8 parental and A1.8 SC^+ ^sorted cells. In contrast, RP.1 CD133^- ^and parental cells had a fivefold increase when compared with the primary RP tumor, whereas CD133^+ ^cells and spheroids retained the expression level found in the original RP tumor (Additional file 8 [panel C]).

Analysis of *Abcg2 *transporter (Additional file 8 [panel D]) in A1.8 cells revealed higher levels in SC^+ ^and parental cells than in SC^- ^or spheroids. RP.1 CD133^- ^cells showed the highest level of *Abcg2*, whereas RP.1 parental cell line, CD133^+ ^fraction, and spheroids showed fivefold increase as compared with levels found in the original RP tumor. These data indicate that expression of *Abcb1*, but not that of *Abcg2*, correlates with drug resistance.

### 17-DMAG sensitizes *Brca1 *cells to chemotherapeutic agents

**Figure 4:**
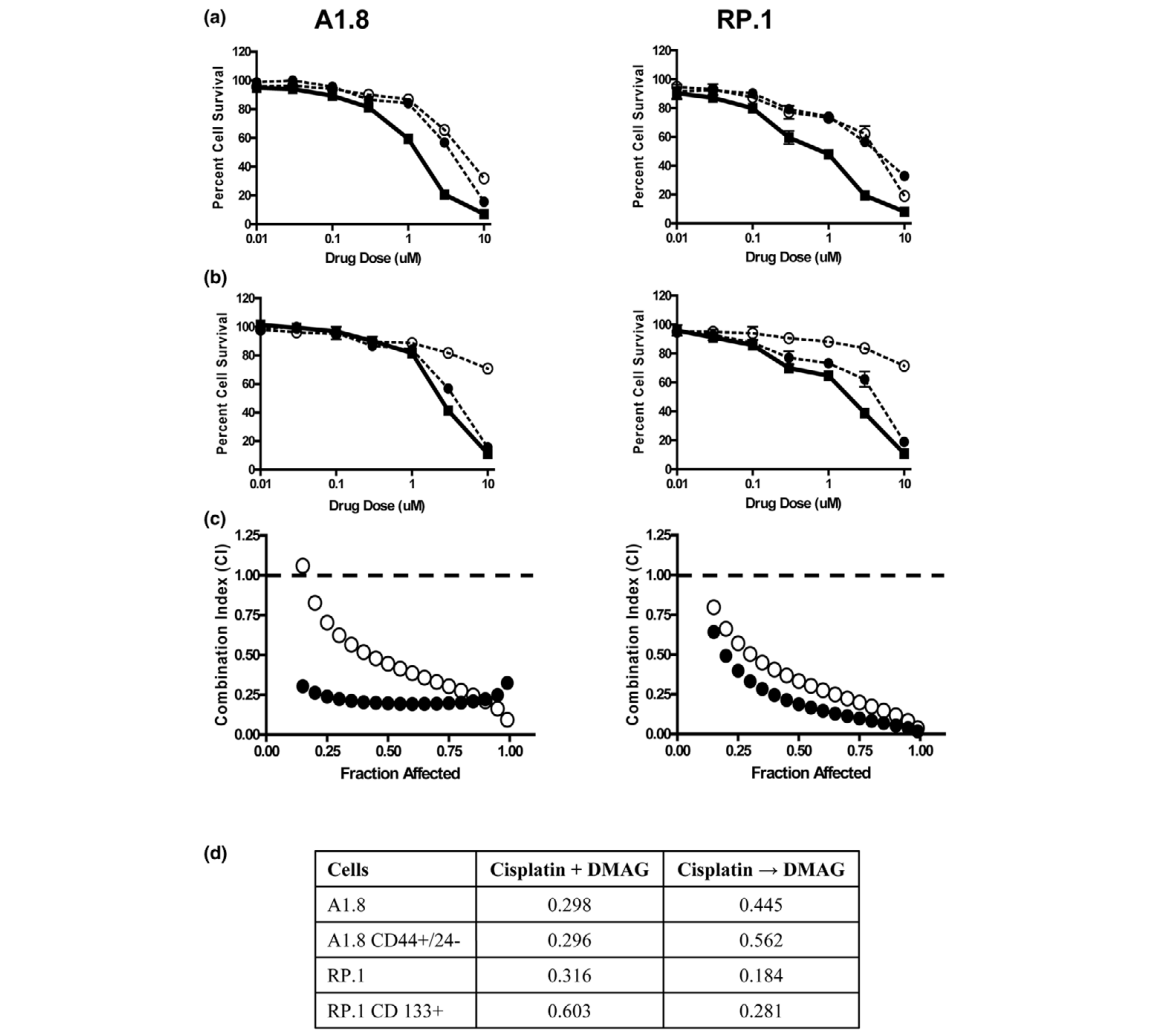
17-DMAG is synergistic with cisplatin for *Brca1 *parental and cancer stem cells. Cells were treated with increasing concentrations of cisplatin, 17-DMAG (17-dimethylaminoethylamino-17-demethoxygeldanamycin hydrochloride), or a combination of both drugs at a constant 1:3 ratio. **(a) **Simultaneous treatment for 48 hours. The open circles and dotted lines represent exposure to 17-DMAG, the filled circles and dotted lines represent exposure to cisplatin as single agents, and the filled squares linked by a solid line show combination. **(b) **Sequential addition of the drugs. The filled circles show exposure for 48 hours to cisplatin, the open circles and dotted lines represent 24 hours pf exposure to 17-DMAG, and the filled squares linked by a solid line show combination. The ordinate indicates concentrations of cisplatin, and the error bars represent ± standatd deviation from sextuplicates wells of one of more than three independent experiments. **(c) **Visual representation of Combination Index (CI) for each combination calculated using CalcuSyn Software. The closed circles represent simultaneous treatment and the open circles represent sequential addition of cisplatin followed by 17-DMAG. Values of CI below 1 indicate synergy, values above 1 represent antagonism, and CI = 1 corresponds to an additive effect. **(d) **17-DMAG sensitizes parental and cancer stem cells to Cisplatin. CI for cisplatin and 17-DMAG added simultaneously (+) or sequentially (→) to parental cells or cells sorted for putative cancer stem cell markers. Data are derived from more than three independent experiments.

We also examined whether the heat shock protein (HSP)90 inhibitor 17-DMAG sensitizes *Brca1 *cells to DNA-damaging agents and whether the schedule of administration plays a significant role. Doxorubicin, cisplatin, or etoposide were added simultaneously or sequentially, with 17-DMAG added 24 hours before or after DNA damaging agents. Calculation of Combination Index (CI) was modeled by CalcuSyn software to establish synergy or antagonism of the drug interaction. A CI of 1 indicates an additive effect whereas under 1 reflects a synergistic effect. For all cell lines and drugs tested, the combination therapy was synergistic and more effective than either drug alone, but no additional synergy was noted if 17-DMAG was added 24 hours after cisplatin as compared with simultaneous treatment (Figure 4). Similar results were obtained using doxorubicin and 17-DMAG (data not shown). In contrast, significant antagonism with CI above 1 was observed when cells were exposed to 17-DMAG before cisplatin (not shown). These data are consistent with previously reported G_1 _arrest induced by HSP90 inhibitors in other cancer cells, which makes them highly resistant to DNA-damaging agents [20].

We also examined whether the combination of cisplatin with 17-DMAG sensitizes the putative cancer stem cells to DNA-damaging agents. Cells from A1.8 and RP.1 parental cells sorted for respective stem cell positive and negative markers were exposed to cisplatin and 17-DMAG simultaneously or sequentially. Combination therapy was highly synergistic for all drug doses, with CI significantly below 1, regardless of simultaneous or sequential (with 17-DMAG added 24 hours later) schedule of administration (Figure 4c,d).

### Tumor-initiating capacity of cells bearing stem cell markers

**Figure 5:**
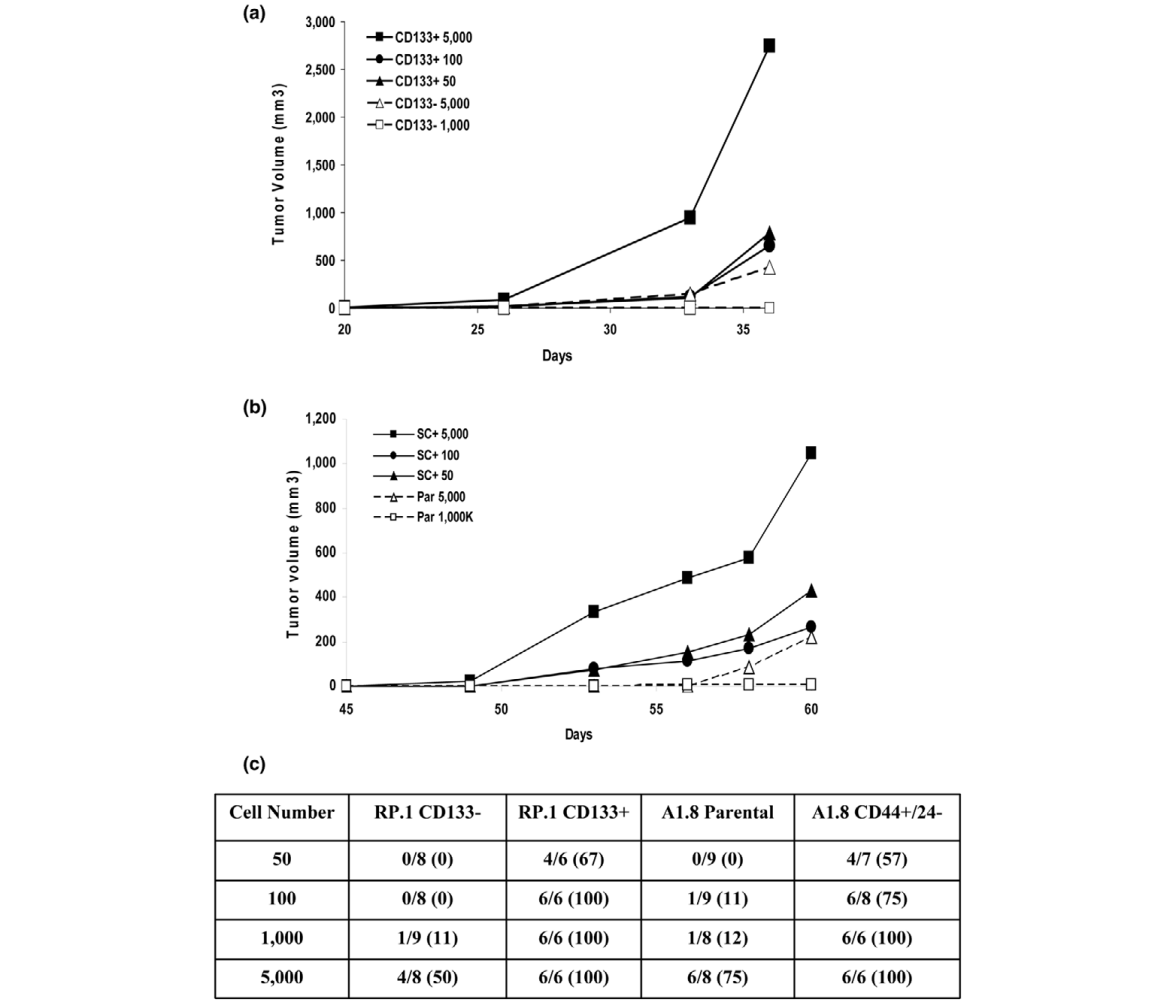
Cells sorted for expression of CD44^+^/CD24^- ^or CD133^+ ^markers are enriched in tumor-initiating cells. Tumor initiating capacity was determined by implantation of 50, 100, 500 or 1,000 cells in mouse fat pad (MFP) #4 of nonobese diabetic/severe combined immunodeficient mice. **(a) **RP.1 CD133^+ ^cells compared with RP.1 CD133^- ^cells, and **(b) **A1.8 CD44^+^/CD24^- ^cells compared with parental cells. Tumor growth rates were monitored, as described in Materials and methods, and average tumor size is shown for each cell type and cell number implanted based on triplicate implantations. **(c) **Total number of mice that grew tumors, as determined from independent experiments performed for each cell line. The percentages of positive tumors are indicated in parentheses.

To determine whether cells that express distinct cancer stem cell markers are tumorigenic *in vivo*, increasing numbers of RP.1 (Figure 5a) and A1.8 (Figure 5b) cells sorted for expression of cancer stem cell markers were injected into mice, starting with 50 cells/injection. Only two out of three mice that received 5 × 10^3 ^CD133^- ^RP.1 cells, and no mice injected with 1 × 10^3 ^cells or fewer formed tumors within 40 days (Figure 5a). In contrast, all cells sorted for the CD133^+ ^marker were highly tumorigenic, with tumors formed by as few as 50 CD133^+ ^cells. In addition, the tumors generated from 5 × 10^3 ^CD133^+ ^cells were at least 10-fold larger that the tumors formed from the same cell number of CD133^- ^cells at the end of 40 days. Animals having no tumors were observed for an additional 60 days. No mouse that received 50 or 100 CD133^- ^cells formed tumors or exhibited evidence of small tumors after examination of the mammary gland after 60 days (not shown). Four of a total of eight mice that received 5,000 cells and an additional three out of nine mice that received 1,000 RP CD133^- ^cells had small tumors, measuring on average less than 200 mm^3^, after 2 months.

Although tumors formed by A1.8 cells grew slower than did RP.1 tumors, A1.8 CD44^+^/CD24^- ^cells were also highly enriched in tumor-initiating cells, and 50 to 100 cells were sufficient to generate tumors in 60 days (Figure 5b). Statistical analysis of multiple experiments conducted in these cells, summarized in Figure 5c, confirms the enrichment of RP.1 CD133^+ ^and A1.8 CD44^+^/CD24^- ^populations in tumor-initiating cells. These *in vivo *tumorigenesis studies for both cell types indicate that *Brca1 *cells bearing distinct cancer stem cell markers are enriched at least 50-fold to 100-fold in tumor-initiating cells.

### Molecular characterization of *Brca1 *cancer stem cells

To determine whether *Brca1 *cells that express putative stem cell markers express other genes that are characteristic of stem cells, we compared RNA levels of stem cell associated genes in cells sorted for positive and negative expression of stem cell markers. Of 84 genes from the SuperArray panel, 11 genes were higher in A.8 CD44^+^/CD24^- ^(SC^+^) than in CD44^-^/24^+ ^(SC^-^) cells, and 18 genes were higher in RP.1 CD133^+ ^than in CD133^- ^cells, as determined by real-time quantitative PCR (Table 1). There was a remarkable overlap among stem cell genes over-expressed in both cell types, with seven shared genes, including *Notch1*, *Fgfr1*, *CD44*, and *Sox1*. Other genes whose over-expression was common between CD44^+^/CD24^- ^and CD133^+ ^cells included *Aldh1a1 *(encoding aldehyde dehydrogenase), which was previously associated with hematopoietic, skin, and CD133^+ ^neuronal stem cells [25]. Genes different between cancer stem cell populations in A1.8 and RP.1 cells included *KRT15*, a marker of basal breast cancer type [26], with 17-fold higher expression in RP.1 CD133^+^, and *Desert hedgehog*, with 4.3-fold higher expression in the A1.8 SC^+ ^than in the SC^- ^population.

**Table 1:** Expression of stem cell genes in cells that express stem cell markers

Gene	A1.8: SC^+^/SC^-^	RP1: CD133^+^/CD133^-^
*T*	>20	4.6
*Sox1*	19.2	2.2
*Aldh1a1*	3.5	3.2
*Fgfr1*	3.2	2.1
*Tert*	1.2	4.4
*Notch1*	2.5	4.1
*CD44*	1.9	3.4
*Ascl2*	7.2	
*Pdx1*	7.1	
*Acan*	6.6	
*Dhh*	4.3	
*ALDH2*		3
*MME*		4.5
*KRT15*		17
*JAG1*		2.2
*FOXa1*		19
*DLL1*		6
*DLL3*		3
*COL9a1*		3
*CDH1*		11
*CD8B*		6
*CCNA2*		2.5

Expression of genes from a panel of 84 stem cell-associated genes was determined by SuperArray real-time PCR using RNA from A1.8 and RP.1 cells sorted for CD44, CD24, or CD133 markers (SC+ and SC-). For a complete list of genes tested, see [48]. The results are expressed as fold increase in cells sorted positive over negative markers from replicate experiments. Data are normalized to five housekeeping genes, as per the manufacturers' instructions. Genes common for both cell lines are presented at the top.

**Figure 6:**
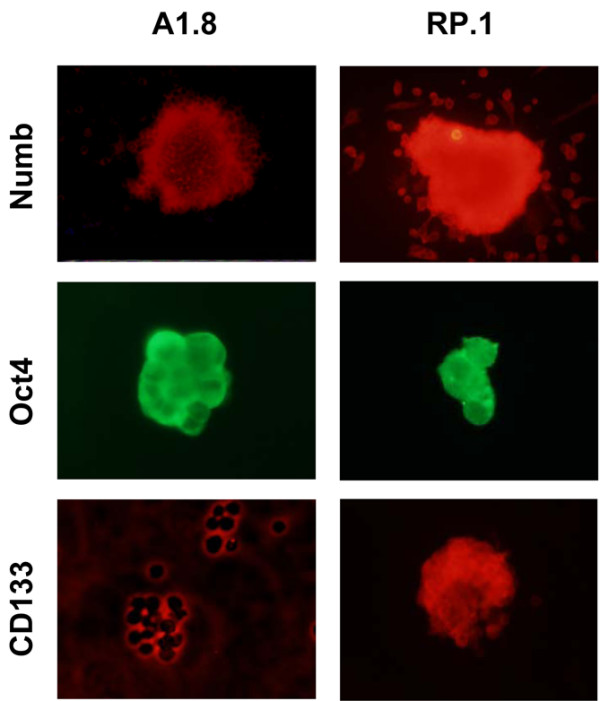
Cells growing as spheroids express stem cell proteins. Immunofluorescence of spheroids formed by A1.8 and RP.1 cells after four passages were stained for Numb, Oct4, and CD133. Cells growing as spheroids were allowed to attach to an eight-well slide, fixed, and stained with indicated antibodies. Staining for Oct4 is visualized in both cell lines, whereas expression of Numb and CD1333 is evident only in RP.1 cells.

To validate our gene expression findings and to define further the two types of cancer stem cells, we conducted immunofluorescence studies on spheroids derived from A1.8 and RP.1 cells using antibodies to common and unique stem cell associated gene products. As seen in Figure 6, spheroids from both cell lines were positive for Oct4, a common key marker of pluripotency. CD133 and Numb, a marker associated with asymmetric division, were present in RP.1 but not in A1.8 spheroids, which confirmed the data obtained by real-time quantitative PCR described above, and both were negative for Nestin, which is a marker of early neuronal differentiation (data not shown). These data suggest that, in spite of having distinct and nonoverlapping cell surface makers, the A1.8 and RP.1 cells have significant similarities in expression of stem cell genes.

We also examined the expression of estrogen receptor 1 in tumors and cell lines and compared it with expression in normal C57BL6 mice virgin mammary gland. The primary tumors and corresponding cell lines exhibited lower estrogen receptor 1 expression, with threefold to fivefold lower expression in A.8 and RP.1, and 22-fold decrease in B.15 cells (Additional file 9 and data not shown). These data are consistent with a previously published report of decreased estrogen receptor expression in fully developed *Brca1 *tumors [27].

## Discussion

Cell lines derived from individual *Brca1 *mouse mammary tumors had distinct and non-overlapping populations of putative cancer stem cell markers CD44^+^/CD24^- ^and CD133^+^. Only cell lines that contained a significant fraction of cells with these markers formed spheroid structures without preliminary sorting, and expansion of these spheroids *in vitro *led to further spontaneous enrichment in cells with stem cell makers. In addition, cells sorted for cancer stem cell markers and cells growing as spheroids were significantly more resistant to chemotherapeutic agents than were parental cells, and were highly tumorigenic in mice. Two conclusions can be derived from these studies. First, *Brca1*-deficient mouse mammary tumors contain heterogeneous populations of cells that share cancer stem cell properties. Second, some *Brca1*-deficient tumors contain CD44^+^/CD24^-/Low ^cells, previously associated with human breast cancer stem cells [10,11,28], whereas others contain CD133^+ ^cells previously associated with tumors in other organs [29]. Which population provides a closer correlate to human disease requires further study.

Human and murine cancer cell lines contain a small fraction of cells that have cancer stem cell properties. These cells are drug resistant, express stem cell associated genes, and have high capacity for reconstituting tumors *in vivo *[9,28,30,31]. Because they constitute a very small cell fraction, their characterization requires isolation, usually by sorting based cell surface markers, and expansion *in vitro*. Our data confirm that cells sorted for cancer stem cell markers reconstitute the parental population after a limited number of passages *in vitro*. Repopulation is indicative of self-renewal, presumably by asymmetric division, and is consistent with stem cell-like properties of these cells. However, the expansion of sorted stem cells *in vitro *results in rapid reduction in the cell fraction that expresses stem cell phenotype, which complicates *in vitro *studies of cancer stem cell biology and development of specific therapies.

Other investigators have reported that cancer-initiating cells sorted from established cell lines or primary tumors form spheroids when plated in semisolid support or serum-free media supplemented with epidermal grwoth factor and basic fibroblast growth factor [9]. Breast cancer-derived spheroid structures have been termed mammospheres and have similarities to those derived from normal mammary glands. Neurospheres derived from neural tissues or gliomas grow in similar conditions [32]. *Brca1 *tumor cells sorted for expression of putative stem cell markers formed spheroids in the absence of attachment without supplementation with growth factors. Furthermore, neither sorting nor growth factor supplementation was required for generation of spheroids from unsorted *Brca1 *cell lines that had a substantial population of cells expressing stem cell markers. The unsorted cells that grew spheroids in long-term culture were significantly enriched in stem cell makers and were highly resistant to DNA-damaging agents after multiple passages *in vitro*. Whether other cancer cells that ordinarily grow in monolayer can survive in the absence of attachment and form spheroids, and become enriched in cancer stem cells remains to be established.

In contrast to the orderly transition of normal stem cells through differentiation with generation of progenitor and terminally differentiated cells, multistep carcinogenesis is likely to generate heterogeneity in cancer-initiating population, which is reflected in different cell surface markers that identify cancer stem cells in different tumor types. Previous studies corroborate that *BRCA1 *deficiency results in genetic instability associated with centrosome amplification, defective cell cycle checkpoint control, and impaired DNA damage repair [33,34]. We found that that same *Brca1*-deficient genetic background gave rise to mammary tumors with two distinct and non-overlapping populations of cells that bear cell surface markers previously assigned to tumor-initiating cells from human breast and other organs. Differences in cancer stem cell populations, which constitute a minute fraction of total tumor, may underlie some of the difficulties in using the genome-wide approach to characterizing molecular profiles for these tumors.

Previous studies showed that 200 CD44^+^/CD24^-/Low ^human breast cancer cells reconstitute the entire population of cancer cells *in vitro *and form tumors *in vivo*, whereas larger numbers of parental cells or cells sorted for the absence of these markers are needed to generate smaller, slower growing tumors [10,11,28]. We found that CD44^+^/CD24^- ^*Brca1*-deficient mouse mammary tumor cells have cancer stem cell characteristics *in vitro*, and 50 of these cells are sufficient to initiate tumors in mice.

CD44 is a complex, multispanning, transmembrane glycoprotein whose expression correlates with drug resistance and poor prognosis in many malignancies [35]. In addition to hyaluronic acid, CD44 binds fibrinogen, fibronectin, collagen, laminin, fibroblast growth factor-2, other heparin-binding growth factors, and osteopontin, an inflammatory cytokine that is associated with metastatic progression. Recent reports suggested heterogeneity in human breast cancer stem cells that express CD44 [36,37]. Whether CD44 expression plays a direct role in drug resistance by activating multiple survival pathways via growth factor receptors or integrin-mediated 'inside-out' signaling is not known and warrants further investigation.

Although expression of CD24 negatively correlated with stem cell characteristics in human breast cancer [10,11,28], the situation is more complex in mouse mammary tumors. In murine mammary gland the CD24^low ^cells correspond to myoepithelial cells and have high mammary fat pad reconstitution capacity, whereas the CD24^+ ^population has low capacity for reconstitution and is devoid of normal mammary stem cells [34]. Furthermore, Weinberg and colleagues [39] recently reported that mammosphere-forming and tumor-initiating capacities reside within CD24^+ ^freshly isolated normal mammary cells, but when these cells are briefly cultured the CD24^- ^population was enriched with cancer stem cells. These previously reported differences in expression of CD24 in different tumor types and in normal mouse mammary epithelium support our search for the role of CD24^- ^cells in combination with a more established marker, CD44, in *BRCA1*-deficient tumors.

Expression of CD133 in cancer-initiating cells is well documented for brain, prostate, and colon cancers [29] but has not been described in breast cancer. We detected 2% to 4% of CD133^+ ^cells in multiple cell lines derived from one *Brca1 *tumor with characteristics similar to those found in CD44^+^/CD24^- ^cells, including drug resistance, the ability to form spheroids with further 30% enrichment in CD133^+ ^cells, expression of stem cell genes, and *in vivo *reconstitution of tumors with as few as 100 cells. CD133, also known as prominin-1, is a cell-surface glycoprotein with five transmembrane domains and two large glycosylated extracellular loops that localize to membrane protrusions [29]. The function of CD133 in cancer stem cells has not been established, but one alternatively spliced form binds cholesterol and thus may be involved in Hedgehog signaling, which is required for primitive cell differentiation and epithelial-mesenchymal interactions [40].

We previously described generation of tumors from cell suspensions from multiple genetically engineered mouse mammary tumors and their expansion by transplantation in naïve recipients *in vivo *[16]. Gene expression analysis of individual *Brca1 *mammary tumors and their subsequent passages *in vivo *revealed substantial heterogeneity in gene expression [40], as predicted by differences in frequency and identity of cancer stem cell populations in cell lines derived from these tumors. Thus, in contrast to other models, such as MMTV-PyMT and MMTV-wnt1, in which pooling individual mouse tumors can generate sufficient material for basic and translational studies [16,42], *Brca1 *tumors will need to be analyzed individually and these studies are limited by the size of each original tumor. Thus, generation of cell lines provided valuable reagents for these studies.

A fundamental characteristic of cancer stem cells is their resistance to chemotherapeutic agents. Much understanding of *BRCA1 *drug resistance comes from studies of a single human cell line, namely H1937, which is null for the *BRCA1 *gene. Although replacement of this gene increases resistance to vinorelbine and cisplatin, it does not change sensitivity to other agents, such as docetaxel [43,44]. This suggests that other mechanisms may determine drug resistance in that cell line. Consistent with these observations, we found that H1937 cells contain a significant (2% to 3%) population of cells expressing CD44^+^/CD24^- ^and no detectable CD133^+ ^(data not shown). The contribution of these cells to drug sensitivity remains to be determined.

Over-expression of several ABC transporters has been linked to drug resistance. Our analysis showed that *Abcb1b *expression correlated with an increase in cells having stem cell markers in A1.8 and RP.1 cell lines. Expression of *Abcb1b *was further enriched in A.8 cells sorted for stem cell markers. However, this did not occur in RP.1 cells, because CD133^- ^cells, but not CD133^+ ^cells, exhibited greater expression of *Abcb1b*. Increased *Abcg2 *expression, previously associated with cancer stem cells, was evident in all *Brca1 *cell lines, regardless of presence or absence of putative stem cell fraction. Furthermore, the highest expression of *Abcg2 *was detected in B.15, a cell line that was not enriched in stem cell markers and did not form spheroids. Thus, expression of other transporters, or different drug resistance mechanisms, such as aldehyde dehydrogenase, which was over-expressed in both CD44^+^/CD24^- ^and CD133^+ ^cell types, may be operational in *Brca1 *cancer stem cells. Aldehyde dehydrogenase-1 participates in oxidation of retinol to all-*trans *retinoic acid, and confers drug resistance to chemotherapeutic agents by an uncertain mechanism [45].

We previously demonstrated schedule-dependent synergy of the HSP90 inhibitor 17-DMAG with doxorubicin for lymphoma cells [20]. Here, we found that addition of 17-DMAG simultaneously or after chemotherapeutic agents sensitizes *Brca1 *cancer stem cells to three types of DNA-damaging agents: doxorubicin, cisplatin, and etoposide. Because HSP90 inhibitors impair multiple signal transduction pathways, resulting in decreased cell survival and DNA repair [20,24,46], our data showed that functional inactivation of *Brca1 *is particularly vulnerable to this combination. Whether HSP90 inhibitors sensitize other cancer stem cells to chemotherapeutic agents remains to be established. Development of cancer stem cell-directed therapies has been hampered by inability to expand cancer stem cells *in vitro*. Enrichment of cancer stem cells in spheroids formed by *Brca1 *cell lines provides ample material for studies of cancer stem cell biology and preclinical testing.

## Conclusion

*Brca1*-deficient mouse mammary tumors harbor heterogeneous cancer stem cell populations, and CD44^+^/CD24^- ^cells also identify human breast cancer stem cells. In addition, long-term spheroid-forming assay may allow rapid screening of tumors for enrichment in cancer stem cells, identification of additional stem cell markers, and development of potentially curative therapies that target the putative cancer stem cell.

## Abbreviations

ABC = ATP-binding cassette; CI = Combination Index; 17-DMAG = 17-dimethylaminoethylamino-17-demethoxygeldanamycin hydrochloride; IC_50 _= 50% inhibitory concentration; MMTV = mouse mammary tumor virus; MTS = 3-(4,5-dimethylthiazol-2-yl)-5-(3-carboxymethoxyphenyl)-2-(4-sulfophenyl)-2*H*-tetrazolium, inner salt; PBS = phosphate-buffered saline; RT-PCR = reverse transcription polymerase chain reaction.

## Competing interests

The authors declare that they have no competing interests.

## Authors' contributions

MHW developed all cell lines, and performed the flow cytometry labeling, RNA extraction and analysis, as well as drug testing *in vitro*. AMC performed all studies on expression of ABC transporters. CDS performed additional cell labeling, some of the *in vivo *injections into mouse fat pad, and tumor measurements. MDC performed tumor measurements and tumor dissections. SVA advised on testing and data interpretation for ABC transporter expression and other drug resistance mechanisms. LV performed tumor dissection, *in vivo *injections of cells into mammary fat pad, overall coordination, and design of the study.

## Supplementary Material

Additional file 1 File providing a list of oligonucleotide primer sequences for the mouse ABC transporters and plasma membrane calcium ATPase 4 (housekeeping gene) for quantitative real-time RT-PCR. Click here for file

Additional file 2 File showing the morphologic appearance of all 16 cell lines developed from five original independent *Brca1 *mammary tumors (A1, B, P3, P2, and RP) grown in monolayer. Click here for file

Additional file 3 File showing that expression of putative stem cell markers (CD44^+^/CD24^- ^and CD133^+^) occurs on distinct and non-overlapping cell populations. (A) A1.8 cells stained simultaneously with antibodies for CD44, CD24, and CD133 (upper panel). The lower panel shows compensated dual staining for CD44/CD133, CD44/CD24, and triple staining for all three markers. Only 0.02% of A1.8 cells express all 3 markers. (B) RP.1 cells were stained and analyzed as above. No cells bearing all three markers are detectable. One of two independent analyses is shown here. Click here for file

Additional file 4 File showing that RP.1 cells growing as spheroids in the absence of attachment are enriched in CD133^+ ^cells. (A) Parental cells and (B) cells dissociated from spheroids after expanding for four passages *in vitro *were stained side-by-side for CD133 and examined by fluorescence-activated cell sorting. The percentage of CD133^+ ^cells is indicated in each box. Note that a distinct CD133^High ^population is now evident in spheroid-derived cells. One of three independent experiments is shown here. Click here for file

Additional file 5 File showing the morphologic appearance of unsorted cells plated in the absence of attachment from six cell lines that represent five individual tumors. A1.1, A1.8, B.15, P3.17, P2.1, and RP.1 cells were grown in 96-well low-binding plates for 2 weeks, dispersed into single cells, and expanded in six-well low-binding plates. One of more than three independent experiments is shown here. Click here for file

Additional file 6 File showing differences in frequency of CD44/CD24 cells in A1.8 cell line that were growing in monolayer as compared to spheroids. (A) Fluorescence-activated cell sorting (FACS) analysis of stem cell markers from unsorted A1.8 parental cells is compared with SC^+ ^(CD44^+^/CD24^-^) and SC^- ^(CD44^-^/CD24^+^) cells sorted by FACS after growing as monolayers in the third passage (P.3). (B) RP.1 parental and CD133^+ ^and CD133^- ^cells sorted and passaged as monolayer twice (P.2) before analysis. One of three independent experiments is shown. Click here for file

Additional file 7 File showing the sensitivity of *Brca1 *cell lines to doxorubicin, cisplatin, and the HSP90 inhibitor 17-DMAG. Cytotoxicity is determined by MTS assay for four representative *Brca1 *cell lines: A1.8, P3.17, B.15, and RP.1. Cells were exposed to increasing concentrations of (A) doxorubicin, (B) cisplatin, and (C) the HSP90 inhibitor 17-DMAG. Percentage survival (± standard deviation from six replicate wells) after 24 hours of exposure to drugs is represented by open symbols and dotted lines, and after 48 hours by solid symbols and lines. The ordinate shows concentrations of individual drugs. One of three independent experiments for each cell type is shown here. Click here for file

Additional file 8 File showing the differences in expression of ABC transporters, *Abcg2 *and *Abcb1*, detected among the cell lines and parental tumors. (A) Expression of *Abcg2 *among six *Brca1 *cell lines. (B) Expression of *Abcb1 *in five cell lines that represent each one of the five independent tumors. Relative (C) *Abcb1 *and (D) *Abcg2 *expression in parental cells, cells sorted for respective stem cell markers, and unsorted cells growing as spheroids. Expression of each transporter is normalized to *Pmca4 *housekeeping gene, as described in Materials and methods. The bars represent ± standard deviation from triplicate samples. One of three independent experiments is shown. Click here for file

Additional file 9 File showing estrogen receptor (ESR)1 expression in individual cell lines and normal mouse mammary gland from 8-week-old C57BL6 mice, as determined by quantitative RT-PCR. The data were calculated using the ΔΔCT method from duplicate samples, in which the expression in each sample run was compared with expression in mammary gland, averaged, and normalized to cyclophilin, which was used as a housekeeping gene. Click here for file
