# Supplementary material for: Brca1 breast tumors contain distinct CD44+/CD24- and CD133+ cells with cancer stem cell characteristics
Source: Breast Cancer Res. 2008 Feb 1;10(1):R10. doi: 10.1186/bcr1855 (PMC2374965; doi:10.1186/bcr1855)
Supplement: Additional file 1 — File providing a list of oligonucleotide primer sequences for the mouse ABC transporters and plasma membrane calcium ATPase 4 (housekeeping gene) for quantitative real-time RT-PCR. [file bcr1855-S1.doc]

**Supplemental Table I. List of oligonucl**eotide primer sequences.

| **ABC transporter** | **Position of primer** | **Forward oligo sequence** | **Reverse oligo sequence** |
| --- | --- | --- | --- |
| Abcb1a | 2409-2564 | TCCTCACCAAGCGACTCCGA | CAGCAAGCCTAGACCCTGTAGC |
| Abcb1b | 2322-2472 | CAAGAGATGATGACCATGAAACTAAACGAC | TATCGGACTCGCTTGGTGAGG |
| Abcc1 | 120-282 | CTTTACCAAGTGCTTTCAGAACACGG | CCACAGAAAGAATCCTAAGGCAGT |
| Abcg2 | 1554-1727 | GCCAGTCTATGTTACCTCTTTCTGTCAC | TGCATTCCAGCGGCATCATATTTCA |
| Pmca4 | 1327-1482 | AAGGACAACAACTTGGTACGGC | GGGCTGTGGGATCTGGCG |
