# Supplementary material for: Brca1 breast tumors contain distinct CD44+/CD24- and CD133+ cells with cancer stem cell characteristics
Source: Breast Cancer Res. 2008 Feb 1;10(1):R10. doi: 10.1186/bcr1855 (PMC2374965; doi:10.1186/bcr1855)

Supplemental Figure 1

Morphology of 16 cell lines under phase contrast microscopy (20x)

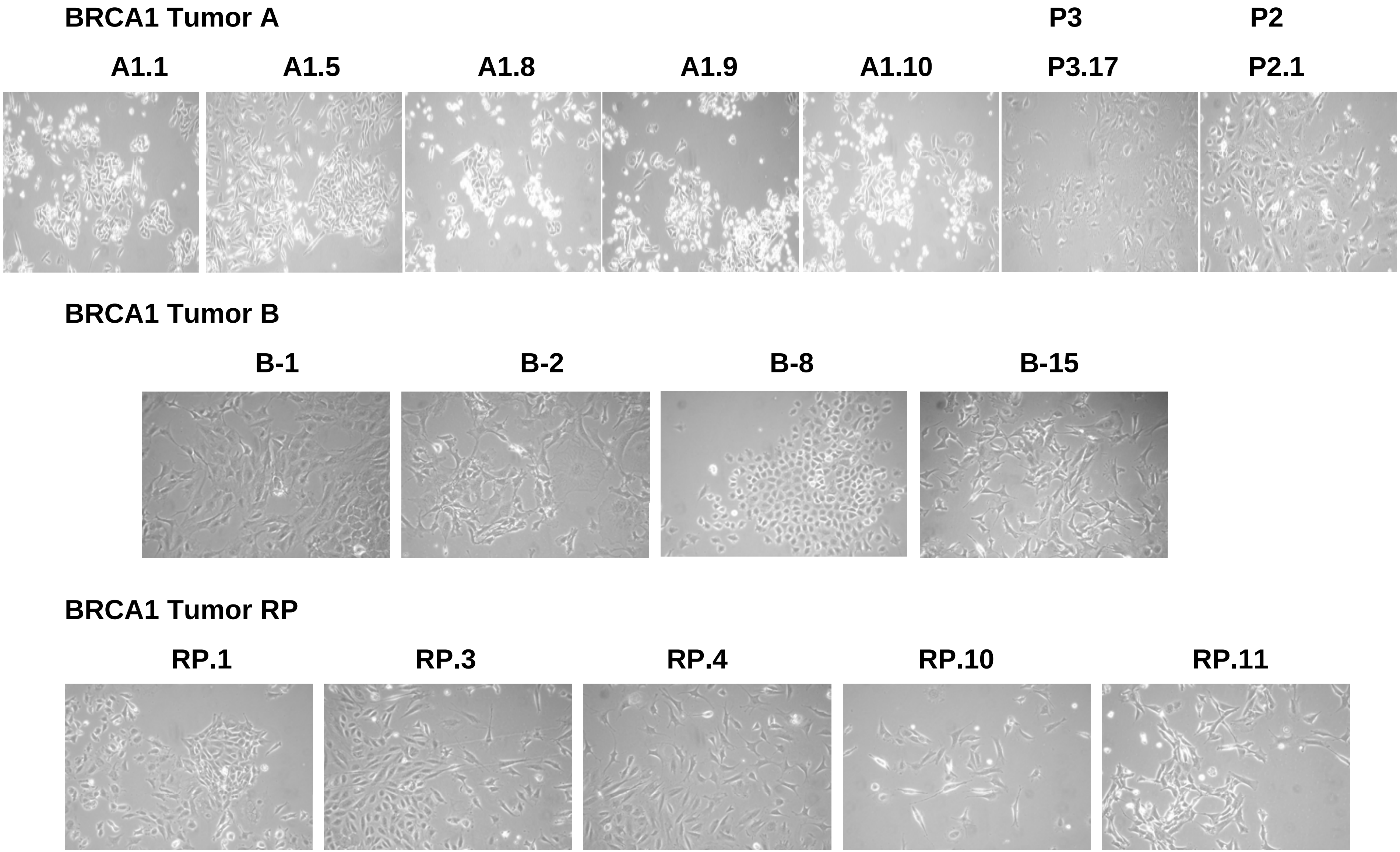

Supplement: Additional file 2 — File showing the morphologic appearance of all 16 cell lines developed from five original independent Brca1 mammary tumors (A1, B, P3, P2, and RP) grown in monolayer. [file bcr1855-S2.pdf]
