# Supplementary material for: Brca1 breast tumors contain distinct CD44+/CD24- and CD133+ cells with cancer stem cell characteristics
Source: Breast Cancer Res. 2008 Feb 1;10(1):R10. doi: 10.1186/bcr1855 (PMC2374965; doi:10.1186/bcr1855)
Supplement: Additional file 3 — File showing that expression of putative stem cell markers (CD44+/CD24- and CD133+) occurs on distinct and non-overlapping cell populations. (A) A1.8 cells stained simultaneously with antibodies for CD44, CD24, and CD133 (upper panel). The lower panel shows compensated dual staining for CD44/CD133, CD44/CD24, and triple staining for all three markers. Only 0.02% of A1.8 cells express all 3 markers. (B) RP.1 cells were stained and analyzed as above. No cells bearing all three markers are detectable. One of two independent analyses is shown here. [file bcr1855-S3.ppt]

## Slide 1
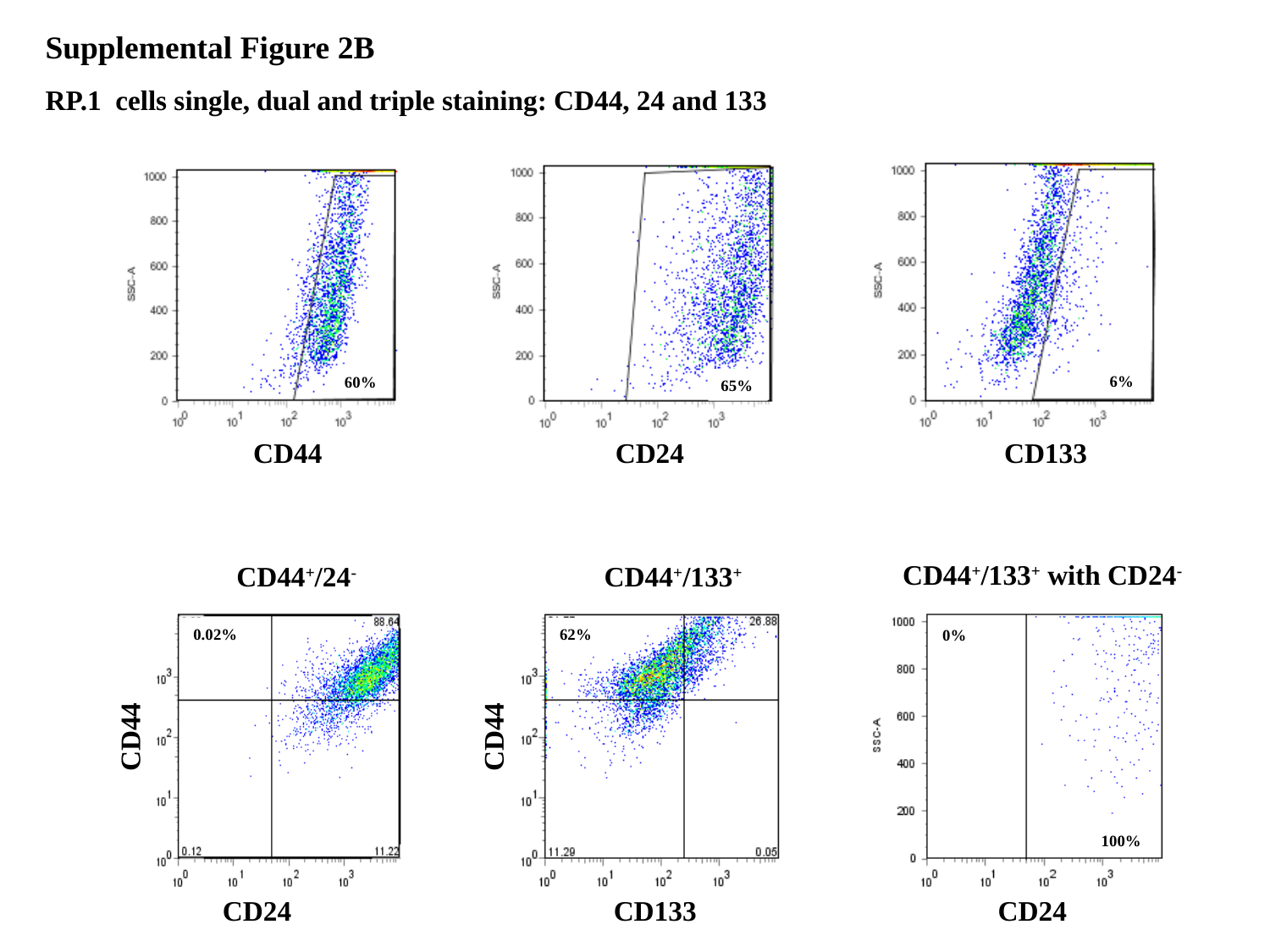

Supplemental Figure 2B
RP.1 cells single, dual and triple staining: CD44, 24 and 133
6%
60%
65%
CD44
CD24
CD133
CD44+/133+ with CD24-
CD44+/24-
CD44+/133+
62%
0.02%
0%
CD44
CD44
100%
CD24
CD133
CD24

## Slide 2
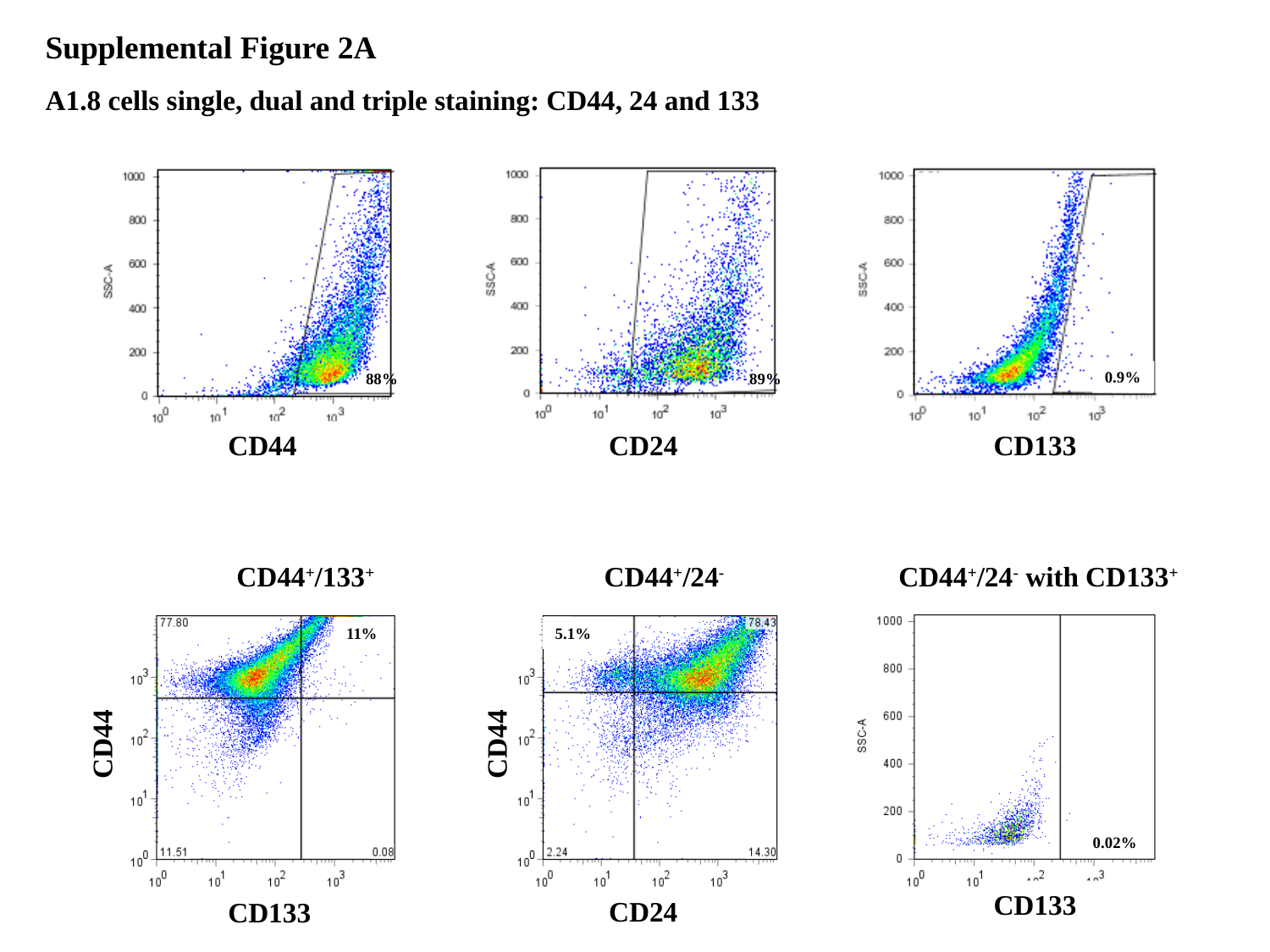

Supplemental Figure 2A
A1.8 cells single, dual and triple staining: CD44, 24 and 133
0.9%
88%
89%
CD44
CD24
CD133
CD44+/133+
CD44+/24-
CD44+/24- with CD133+
11%
CD44
CD133
5.1%
CD44
CD24
0.02%
CD133
