# Supplementary figures and images for: Brca1 breast tumors contain distinct CD44+/CD24- and CD133+ cells with cancer stem cell characteristics
Source: Breast Cancer Res. 2008 Feb 1;10(1):R10. doi: 10.1186/bcr1855 (PMC2374965; doi:10.1186/bcr1855)

## Slide 1
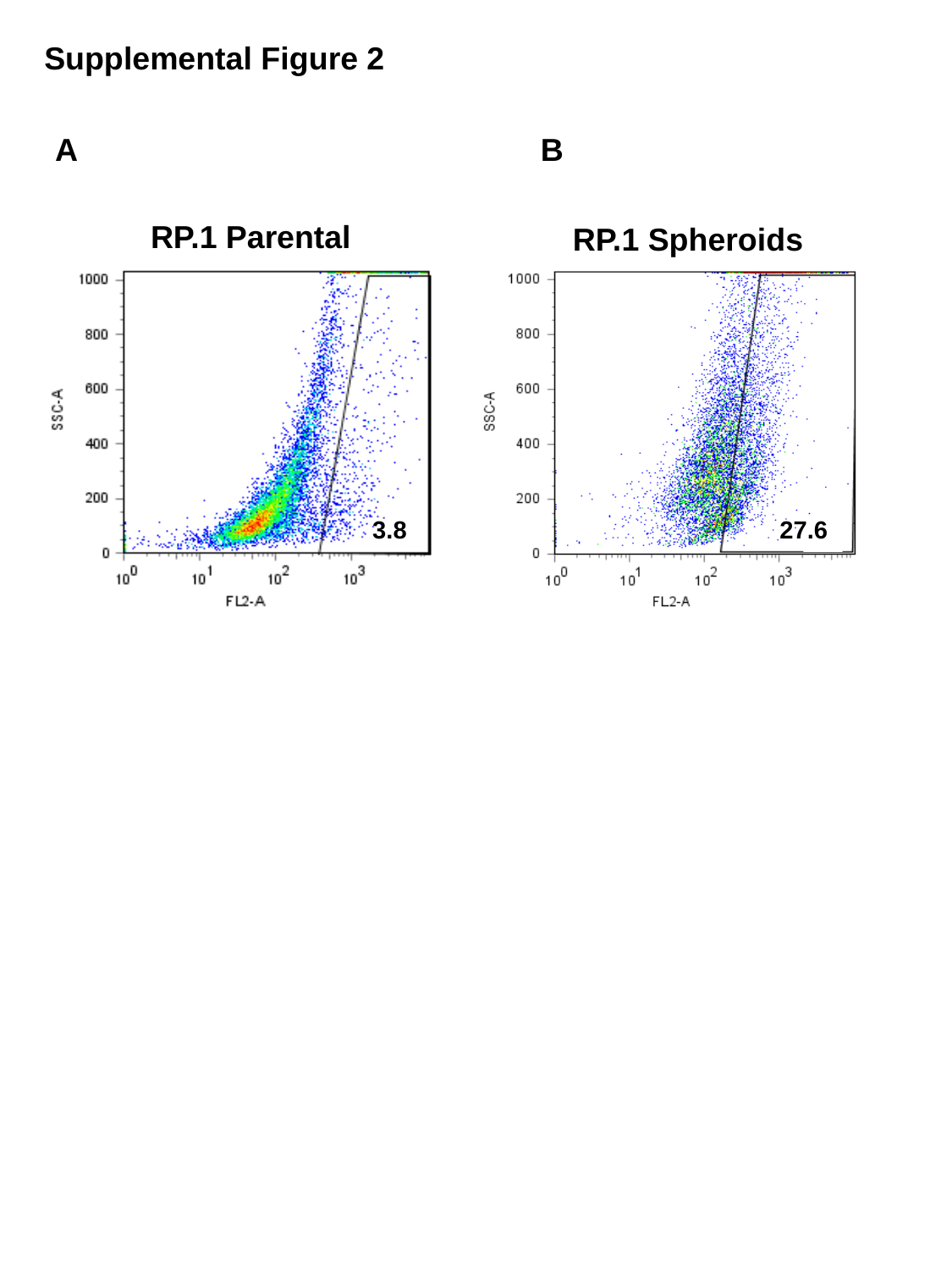

Supplemental Figure 2
A
B
RP.1 Parental
3.8
RP.1 Spheroids
27.6

Supplement: Additional file 4 — File showing that RP.1 cells growing as spheroids in the absence of attachment are enriched in CD133+ cells. (A) Parental cells and (B) cells dissociated from spheroids after expanding for four passages in vitro were stained side-by-side for CD133 and examined by fluorescence-activated cell sorting. The percentage of CD133+ cells is indicated in each box. Note that a distinct CD133High population is now evident in spheroid-derived cells. One of three independent experiments is shown here. [file bcr1855-S4.ppt]

## Supplemental Figure 4

**A1.1**

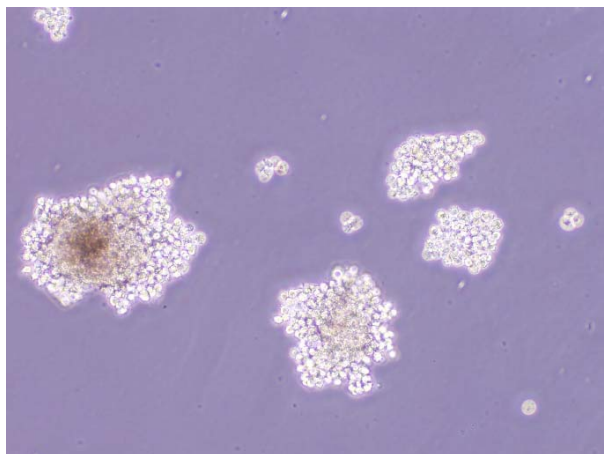

**A1.8**

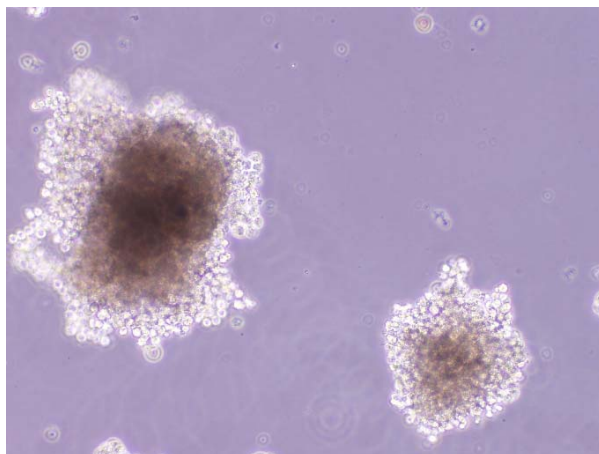

**B.15**

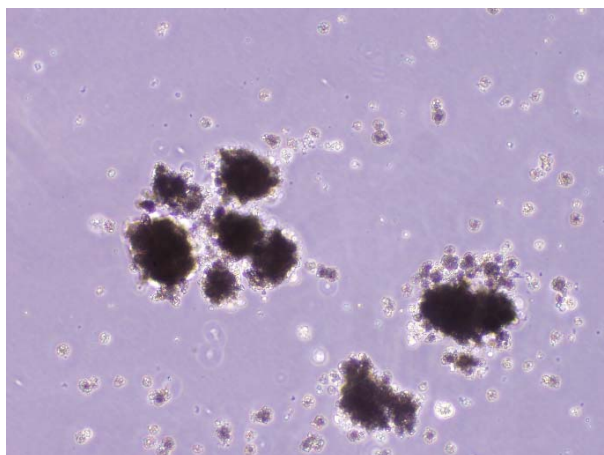

**P3.17**

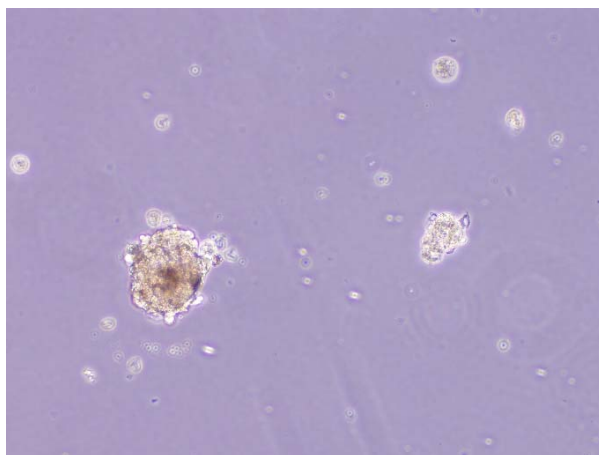

**P2.1**

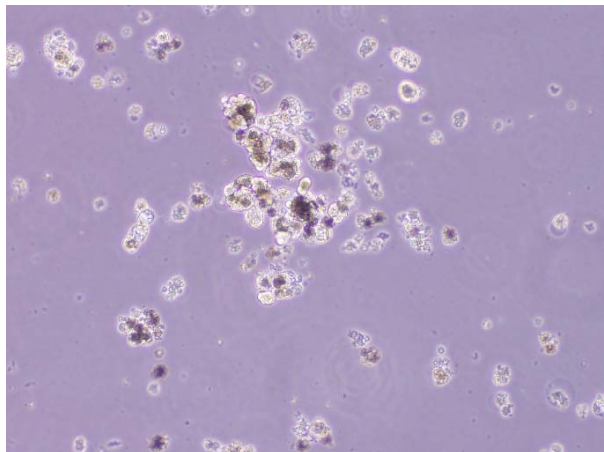

**RP.1**

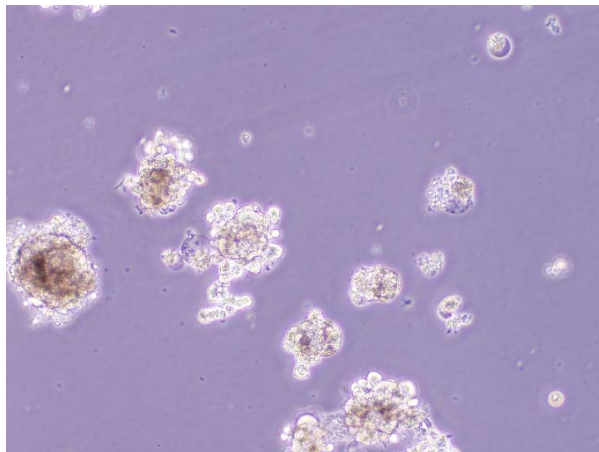

Supplement: Additional file 5 — File showing the morphologic appearance of unsorted cells plated in the absence of attachment from six cell lines that represent five individual tumors. A1.1, A1.8, B.15, P3.17, P2.1, and RP.1 cells were grown in 96-well low-binding plates for 2 weeks, dispersed into single cells, and expanded in six-well low-binding plates. One of more than three independent experiments is shown here. [file bcr1855-S5.pdf]

## Slide 1
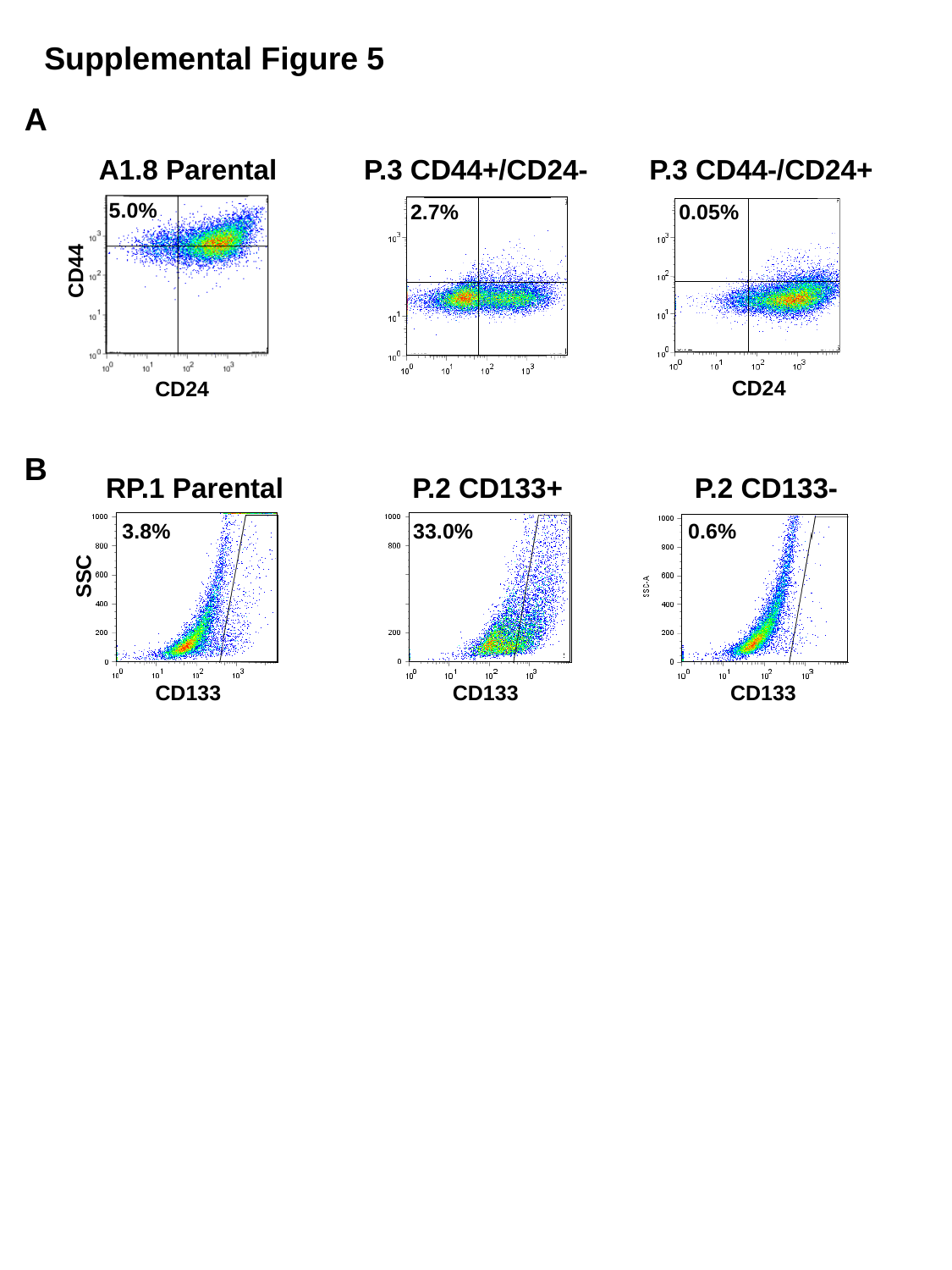

Supplemental Figure 5
A
A1.8 Parental
P.3 CD44+/CD24-
P.3 CD44-/CD24+
5.0%
CD44
CD24
2.7%
0.05%
CD24
CD24
B
RP.1 Parental
P.2 CD133+
P.2 CD133-
3.8%
33.0%
0.6%
SSC
CD133
CD133
CD133

Supplement: Additional file 6 — File showing differences in frequency of CD44/CD24 cells in A1.8 cell line that were growing in monolayer as compared to spheroids. (A) Fluorescence-activated cell sorting (FACS) analysis of stem cell markers from unsorted A1.8 parental cells is compared with SC+ (CD44+/CD24-) and SC- (CD44-/CD24+) cells sorted by FACS after growing as monolayers in the third passage (P.3). (B) RP.1 parental and CD133+ and CD133- cells sorted and passaged as monolayer twice (P.2) before analysis. One of three independent experiments is shown. [file bcr1855-S6.ppt]

## Slide 1
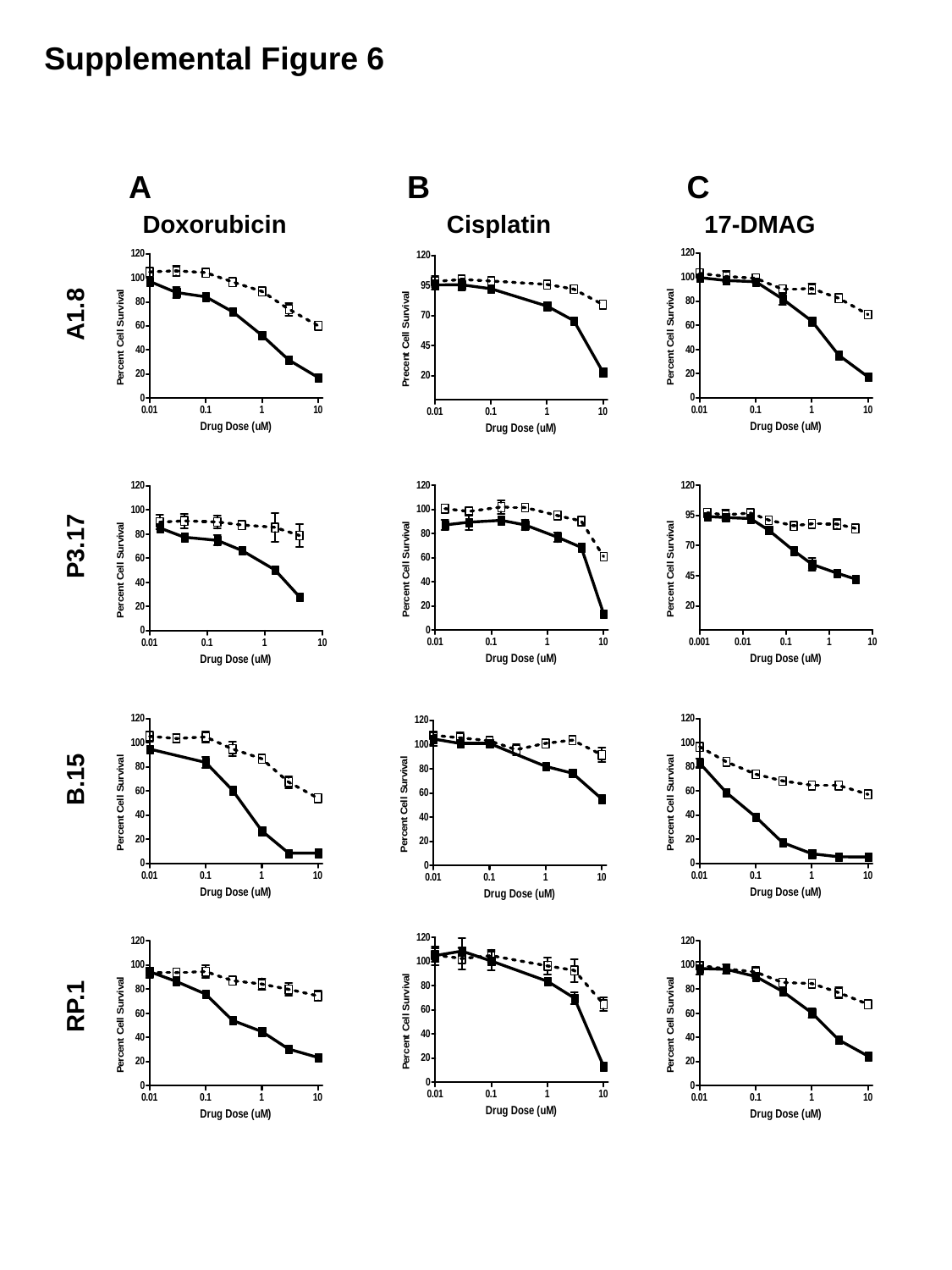

Supplemental Figure 6
A B C
 Doxorubicin Cisplatin 17-DMAG
 RP.1 B.15 P3.17 A1.8

Supplement: Additional file 7 — File showing the sensitivity of Brca1 cell lines to doxorubicin, cisplatin, and the HSP90 inhibitor 17-DMAG. Cytotoxicity is determined by MTS assay for four representative Brca1 cell lines: A1.8, P3.17, B.15, and RP.1. Cells were exposed to increasing concentrations of (A) doxorubicin, (B) cisplatin, and (C) the HSP90 inhibitor 17-DMAG. Percentage survival (± standard deviation from six replicate wells) after 24 hours of exposure to drugs is represented by open symbols and dotted lines, and after 48 hours by solid symbols and lines. The ordinate shows concentrations of individual drugs. One of three independent experiments for each cell type is shown here. [file bcr1855-S7.ppt]

## Slide 1
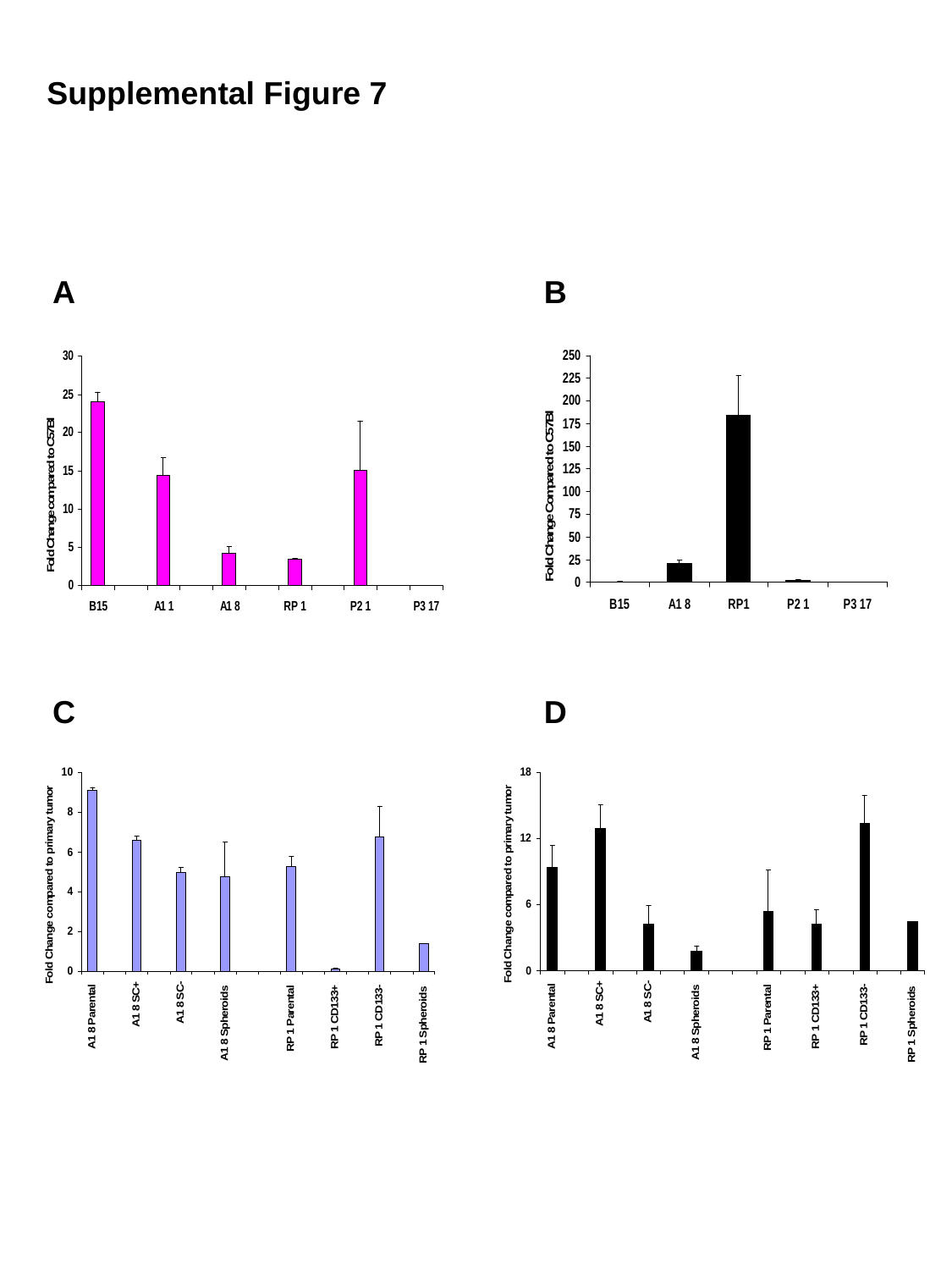

Supplemental Figure 7
A
B
C
D

Supplement: Additional file 8 — File showing the differences in expression of ABC transporters, Abcg2 and Abcb1, detected among the cell lines and parental tumors. (A) Expression of Abcg2 among six Brca1 cell lines. (B) Expression of Abcb1 in five cell lines that represent each one of the five independent tumors. Relative (C) Abcb1 and (D) Abcg2 expression in parental cells, cells sorted for respective stem cell markers, and unsorted cells growing as spheroids. Expression of each transporter is normalized to Pmca4 housekeeping gene, as described in Materials and methods. The bars represent ± standard deviation from triplicate samples. One of three independent experiments is shown. [file bcr1855-S8.ppt]

## Slide 1
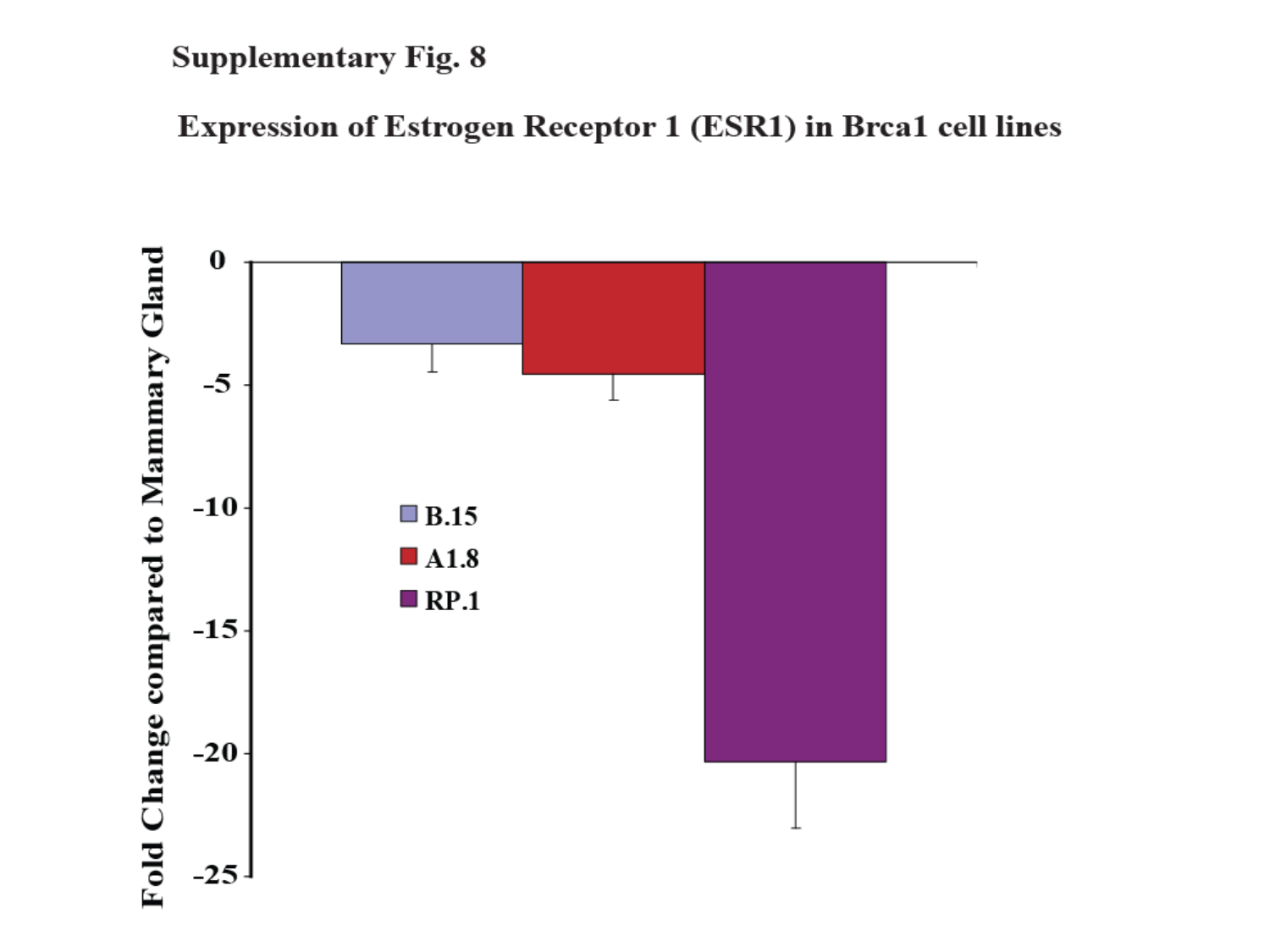

Supplement: Additional file 9 — File showing estrogen receptor (ESR)1 expression in individual cell lines and normal mouse mammary gland from 8-week-old C57BL6 mice, as determined by quantitative RT-PCR. The data were calculated using the ΔΔCT method from duplicate samples, in which the expression in each sample run was compared with expression in mammary gland, averaged, and normalized to cyclophilin, which was used as a housekeeping gene. [file bcr1855-S9.ppt]
